# Supplementary material for: Representation of ethological events by basolateral amygdala neurons
Source: Cell Rep. 2022 Jun 7;39(10):110921. doi: 10.1016/j.celrep.2022.110921 (PMC9638002; doi:10.1016/j.celrep.2022.110921)
Supplement: Document S1. Figures S1–S11 [file mmc1.pdf]

Cell Reports, Volume *39*

Supplemental information

Representation of ethological events by basolateral amygdala neurons

Cristina Mazuski and John O'Keefe

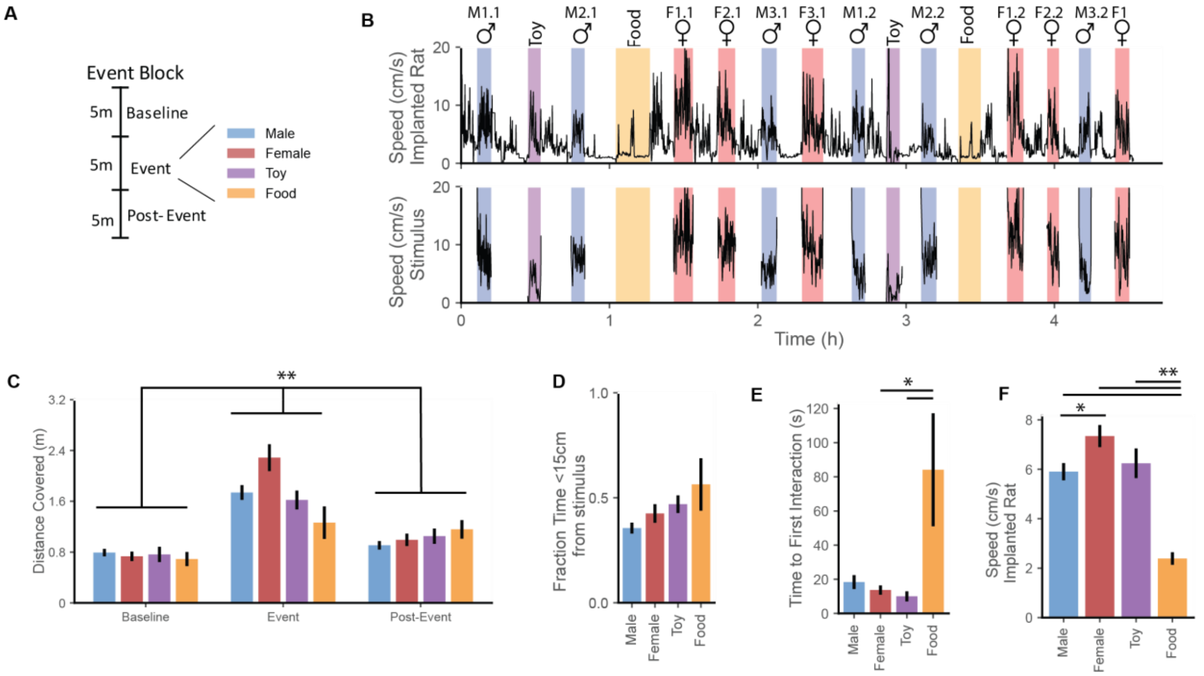

Fig S1. Presentation of a battery of social and non-social stimuli and resulting behavior. Related to Figure1.

(A) Representation of a single event block and different stimuli or event-types presented. During a recording session (representative example in B), an implanted rat was presented with up to 16 different event blocks. The implanted rat was continuously tracked throughout the entire recording session and non-stationary stimuli (male conspecifics, female conspecifics, moving toy) were tracked when present. C) The total distance covered by the implanted rat increases during interaction with stimuli, but there are no consistent differences between different types of stimuli. (**$p<0.001$ for time, $p=0.08$ for event-type, repeated measures 2-way ANOVA) D) Implanted rats spend similar amounts of time within 15cm of stimuli indicating that there was not an overall preference for one type of stimuli over another. ($p = 0.16$; 0.36 ± 0.03, 0.42 ± 0.04, 0.47 ± 0.04, 0.56 ± 0.12; Kruskal-Wallis test) E) Rats take longer to approach and interact with food than moving stimuli (*$p<0.05$; 18.39 ± 4.05, 13.7 ± 2.7, 10.01 ± 2.95, 84.10 ± 33.1; Kruskal-Wallis test). F) There are large differences in speed when the rat is interacting with stationary versus non-stationary stimuli (food vs. males, females or moving toy, **$p<0.01$,). Within the different non-stationary stimuli, the implanted rat moves at slightly higher speeds when interacting with female vs. male conspecifics (*$p<0.05$), but speed during interaction with a moving toy is not different to speed during social interaction (toy vs. male, $p=0.96$; toy vs. female $p=0.42$; 5.90 ± 0.35, 7.34 ± 0.45, 6.24 ± 0.60, 2.40 ± 0.25; Ordinary 1-way ANOVA). Values for plots D-F from male (N=27), female (N=17), toy (N=8), and food (N=8) events respectively.

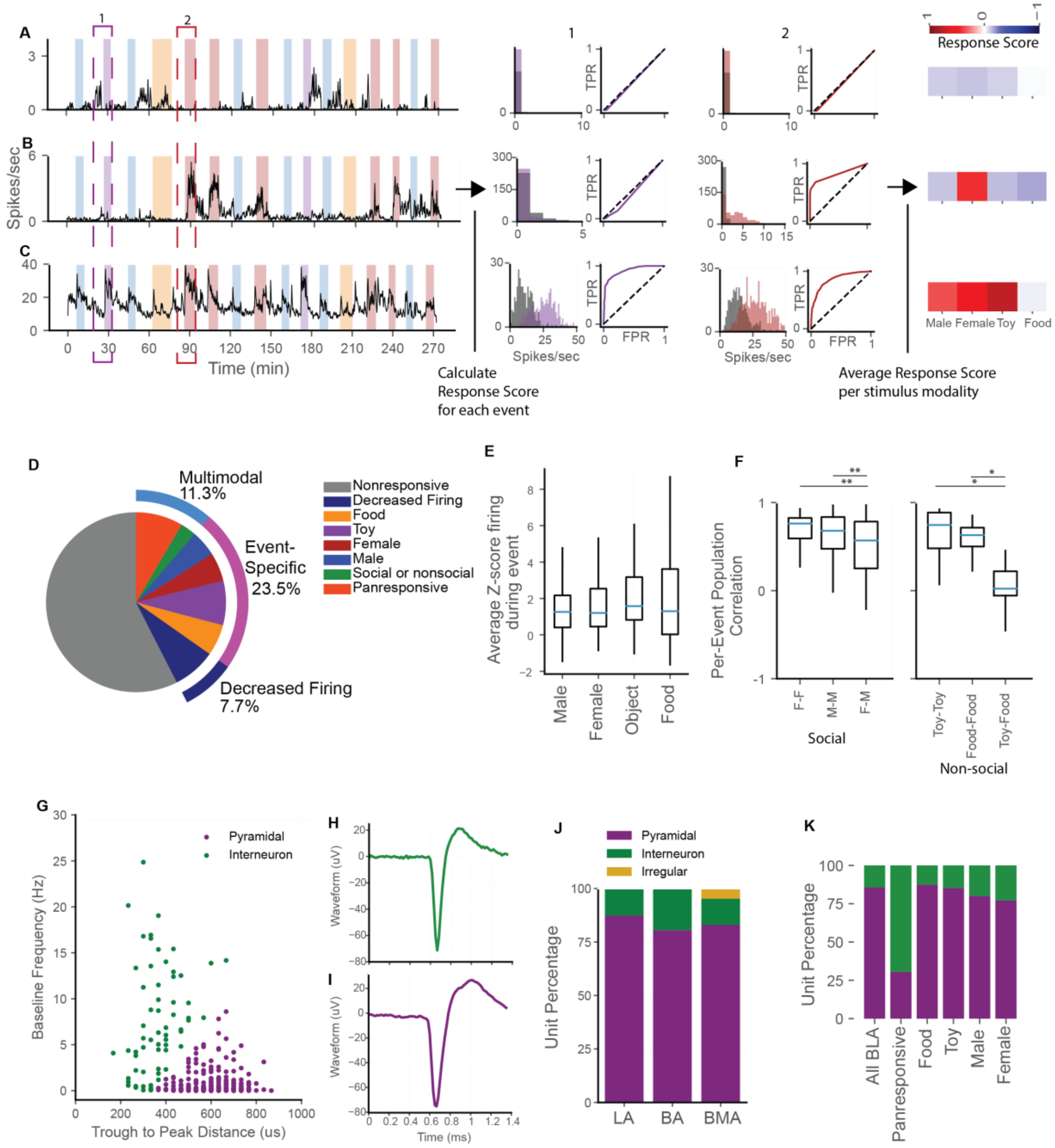

Fig. S2. Calculation of response scores for presentations of different stimuli and neuronal properties. Related to Figure1.

For each neuron, we first computed the receiver operating characteristic curve (ROC curve) and calculated the area under the curve (auROC, between 0-1). We averaged together the auROCs for each event (male, female, toy, food) and converted this into a response score (between -1 and 1). Neurons were classified based on their response scores on each of the four events. A) A representative unresponsive neuron that does not have a positive response to any event. B) A representative event-specific neuron, in this case firing only to females. These neurons show a positive response score to only one event. C) A representative panresponsive neuron, which

shows increases in firing to both social (male/female) and non-social (toy/food) stimuli. Dashed boxes show the periods over which the ROC was calculated for the toy and the first female events. D) The percentage of all BLA neurons with consistent responses to different events. E) There were no differences in the average z-scored firing activity of responsive neurons across different event types. ($p = 0.40$; Ordinary one-way ANOVA; N = 306, 231, 134, 109 trials for male, female, toy and food respectively). F) Population correlation between social (** $p< 0.01$; N = correlation of paired social events, 30, 79, 112 for F-F, M-M and F-M, respectively; Kruskal-Wallis) and non-social (*$p<0.05$; N = correlation of paired non-social events, 4, 4, 16 for toy-toy, food-food, toy-food respectively, Kruksal-Wallis). G) Neurons were classified using K-Means clustering as putative pyramidal or putative interneurons based on their baseline firing frequency (first 5 min of the recording) and the distance between the trough and peak waveform. A representative putative interneuron is shown in (H) and a representative pyramidal neuron shown in (I). J) Small regional differences existed between different BLA nuclei with fewer interneurons being present in the LA than the BA/BMA. Irregular waveforms (where the only waveform peak precedes the waveform trough) were only present in the BMA and were excluded from the analysis in (G). K) Panresponsive neurons are more likely to be putative interneurons than other event types.

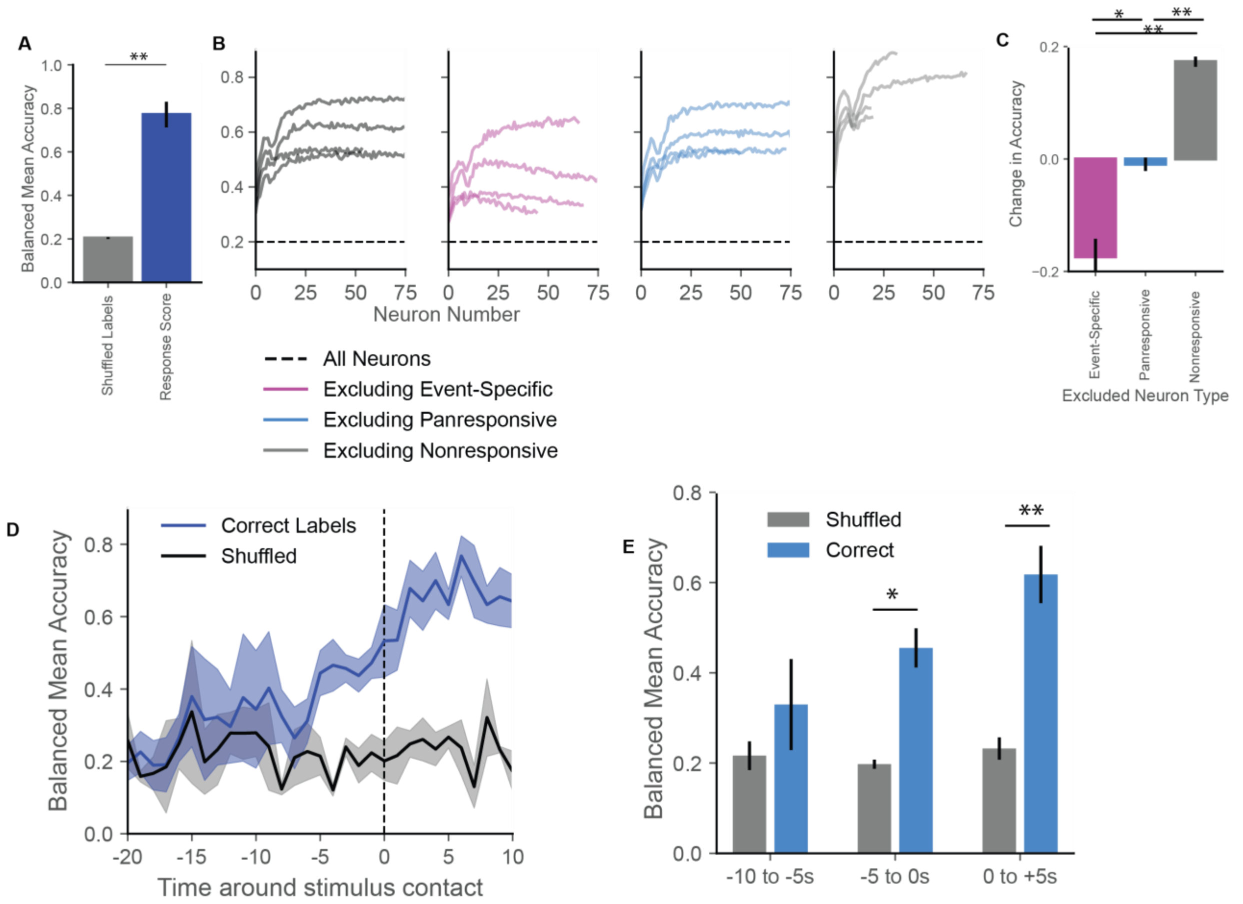

Fig. S3. Reliable decoding of stimulus identity. Related to Figure1.
A) Stimulus identity can be reliably decoded from neuronal response scores using LDA analysis. (** $p<0.01$; 0.20 ± 0.004, 0.78 ± 0.08 for shuffled versus actual, mean ± SEM; N = 4 rats, Student's paired t-test) B) Relatively few neurons are needed for reliable stimulus identity decoding. By excluding specific neuron types (event-specific, panresponsive or nonresponsive) we can measure the relative contribution of each neuron class (each line represents a separate rat, see methods). C) Event-specific neurons carry more information about stimulus identity than panresponsive neurons, which in turn carry more information than nonresponsive neurons. Removing event-specific neurons impairs performance, while removing nonresponsive neurons improves performance (* $p < 0.05$, ** $p < 0.01$; -0.17 ± 0.03, -0.01 ± 0.01, 0.17 ± 0.01 for excluding event-specific, panresponsive or nonresponsive neurons respectively, mean ± SEM; N = 4 rats; repeated measures one-way ANOVA). D-E) To determine whether contact with a given stimulus affected decoding of stimulus identity, we reran LDA using mean firing frequency over 10s around the start of direct contact with the stimulus. Trial identify could be reliably decoded from neuronal activity starting from approximately 5s before direct contact with the stimulus. (-10 to -5s: $p=0.52$; -5 to 0s: * $p<0.05$; 0 to +5s: ** $p< 0.01$; repeated measures 2-way ANOVA, N = 4 rats)

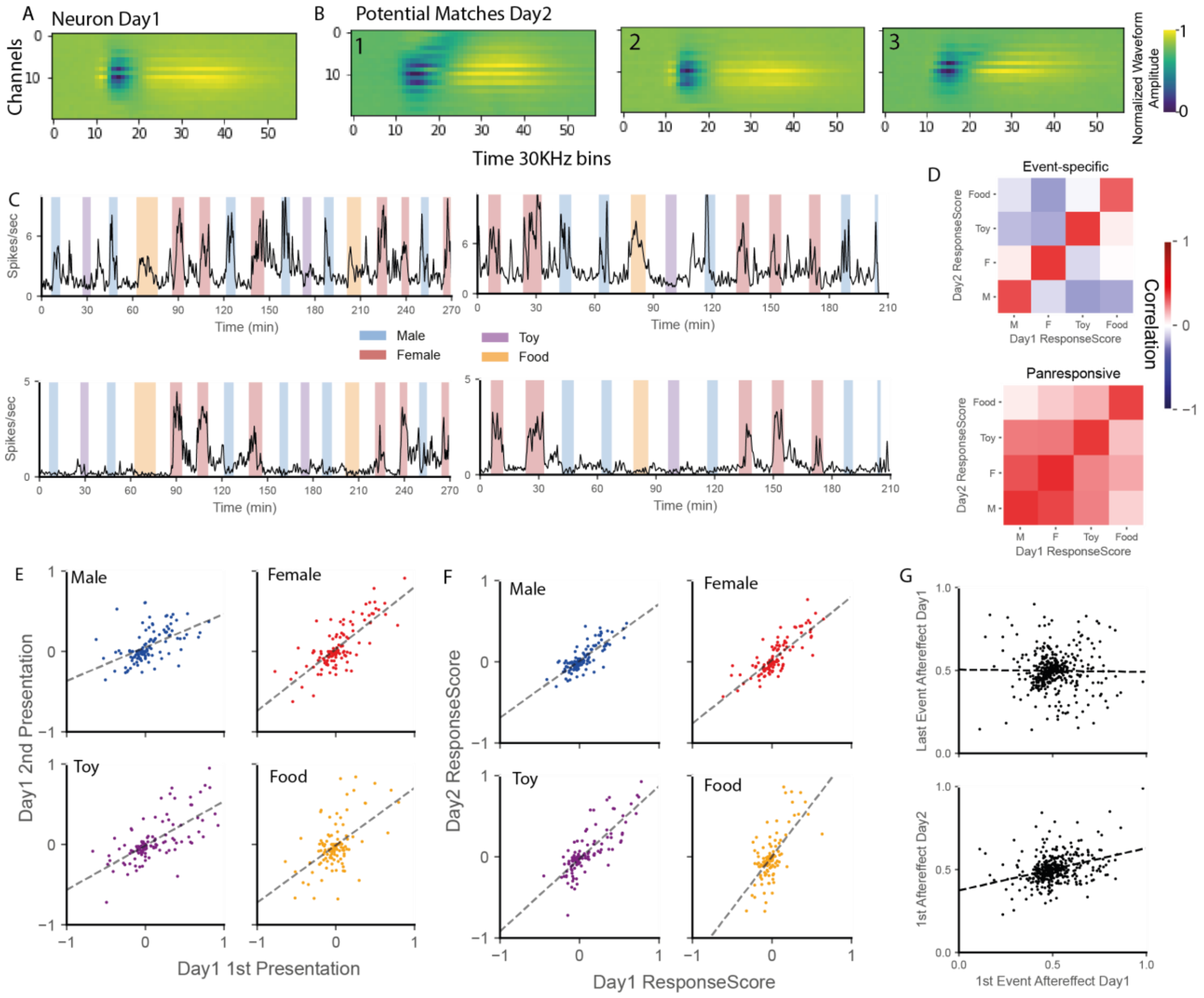

Fig. S4. Stable representation of ethological stimuli across two days. Related to Figure1. 121 out of 426 neurons were tracked across two days of recording. A) We compared the normalized waveform across multiple Neuropixels channels from day1 of the recording to potential matches on day2 (B, see methods). In the example in A-B, the neuron on day1 is ultimately paired with neuron2 on day2. C) Representative examples of a panresponsive (above) and event-specific neuron (below) showing stable coding of different event-types across two days. D) The population vector correlation of the response scores on day1 and day2 for event-specific and panresponsive neurons show stable representation. E) Individual neuronal response scores for single event presentations are highly correlated within a single day of recording (**$p<0.01$; r^2 = 0.52, 0.77, 0.68, 0.54 for male, female, toy and food respectively; N = 121 neurons) F) Neuronal response scores per event-type are highly correlated between 2 days of recording (**$p<0.01$; r^2 = 0.79, 0.82, 0.79, 0.66 for male, female, toy and food respectively; N = 121 neurons). G) Aftereffect strength is not correlated between first and last event presentations within a single day (top panel; within-day aftereffect correlation: p=0.78, r^2=-0.01), but shows correlation across 2 days of recording (bottom panel; across two days aftereffect correlation: **$p<0.01$, r^2 = 0.34). N=426 aftereffect auROC values for all event-types.

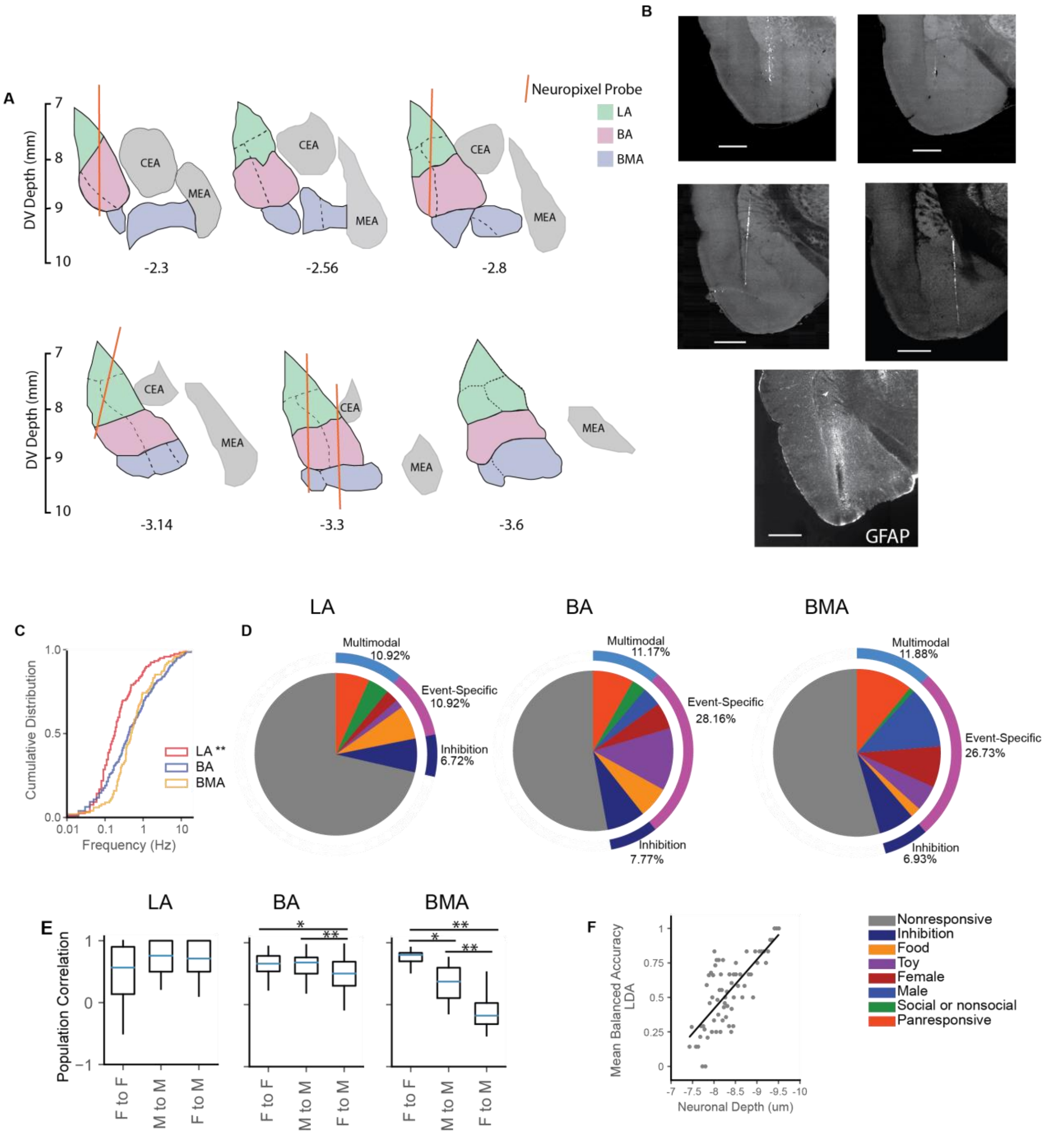

Fig. S5. Histological Reconstruction of Neuropixel probe implants. Related to Figure1. A) Neuropixel tracts were manually registered to a Paxinos rat brain atlas to determine which probe channels were located in specific BLA nuclei. B) Histological images from 5 rats used in this study showing the Neuropixel probe entering the BLA either using the Di-O tract or GFAP staining. (N=119, 206, 101 neurons for LA, BA, and BMA respectively, scale bar = 1mm) C) LA has lower baseline firing rates than the BA and BMA. (**$p<0.01$ LA vs. BA and BMA; 0.76 ± 0.18, 2.0 ± 0.31, 1.4 ± 0.24, baseline firing rate from LA, BA and BMA neurons, respectively, mean ± SEM; Kruskal-Wallis test). This is consistent with the presence of fewer interneurons in that region (S2). D) LA (left) has fewer responsive neurons than BA (middle) and BMA (right)

and neuronal selectivity to specific event-types varies across region with more non-social responses (i.e. event-specific food or toy) in the BA and more social responses in the BMA (i.e. event-specific male or female). E) Discrimination between male and female conspecifics is present in the BA and BMA but not in the LA (LA, $p = 0.62$; ordinary one-way ANOVA; BA, $*p < 0.05$, $**p < 0.01$; ordinary one-way ANOVA, BMA $*p < 0.05$, $**p < 0.01$; Kruskal-Wallis test, N(LA) =36, 12, 32, N(BA) = 24, 58, 84, N(BMA) = 15, 15, 36 for event-pairs F-F, M-M and F-M, respectively). F) Decoding of social stimulus identity changes along the dorsal-ventral axis, with better discrimination of social conspecifics at deeper co-ordinates. (accuracy of decoding social events, $**p < 0.01$, $r^2=0.56$, pearson correlation).

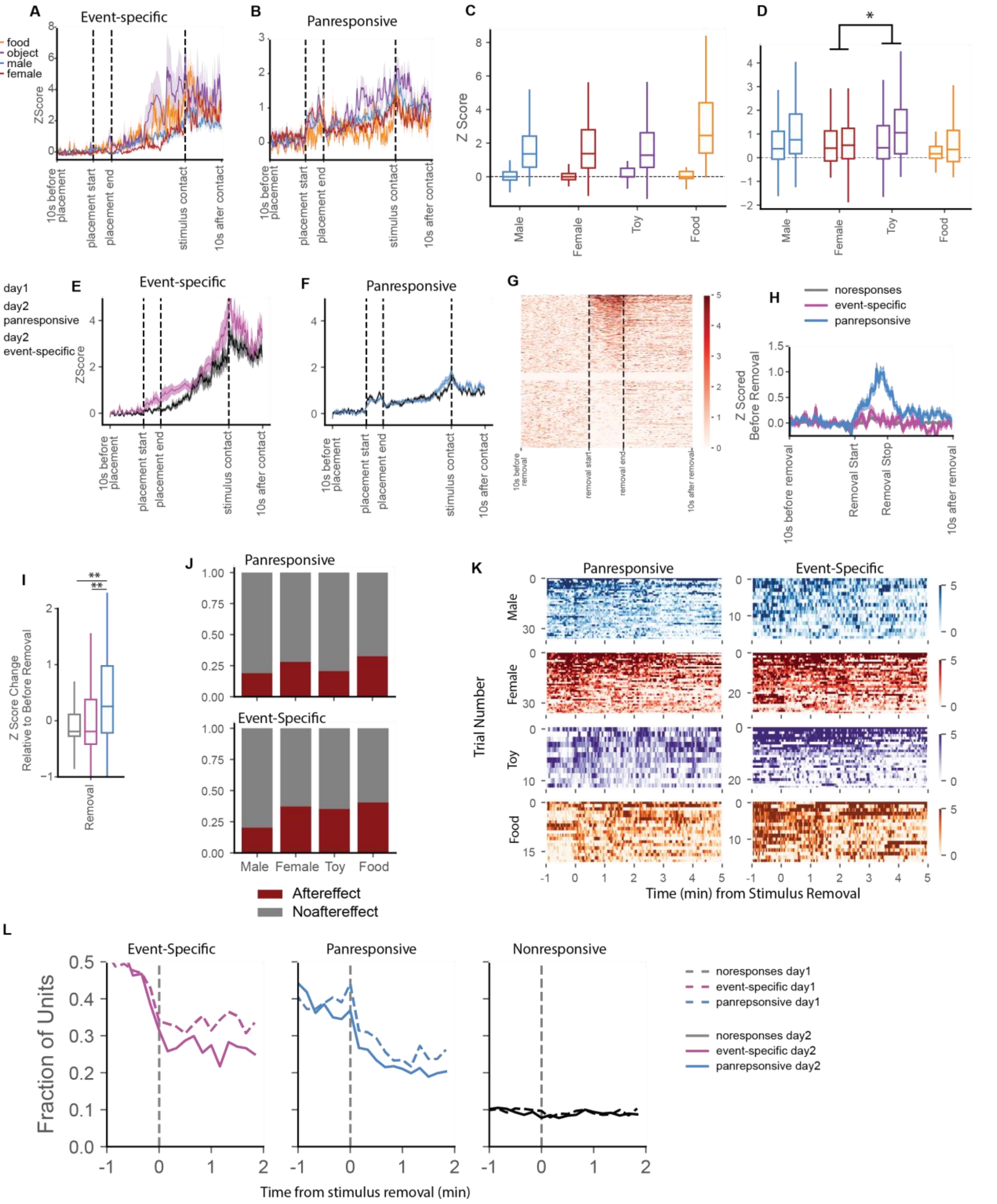

Fig. S6. Similarities in firing onset between different events and across different recording days. Related to Figure2.

A) Event-specific and B) panresponsive neurons show similar time-courses irrespective of the stimulus identity, suggesting that the increases in firing are not due to the presence of a specific sensory cue but to occurrence of the event. The total level of activation during the stimulus presentation and stimulus contact (left and right boxplot for each modality, respectively) was

similar across all stimulus modalities in C) event-specific ($p=0.08$ for event-type differences, $^{**}p<0.01$ for differences across time, N = 222, 174, 152, 92 for event-specific male, female, toy and food responses, respectively, repeated measures 2-way ANOVA) and D) panresponsive neurons, with the exception of slightly higher firing within panresponsive neurons in response to toy stimuli compared to female conspecifics ($^{*}p<0.05$ for event-type differences, $^{**}p<0.01$ for time differences, N = 390, 232, 116, 108 for panresponsive trials during male, female, toy and food events, respectively, repeated measures 2-way ANOVA) E and F) There were no differences in the timecourse in event-specific or panresponsive neurons across the two recording days. G) Z-scored firing data from 10s before stimulus removal to 10s after removal. Data were timewarped between removal start and removal stop to enable comparison between different trials. H-I) Only panresponsive neurons show a transient increase in firing during the removal of the stimulus ($^{**}p<0.01$, N = 1239, 280, 389 for nonresponsive, event-specific and panresponsive trials, respectively; Kruskal-Wallis). J) The proportion of panresponsive and modality-specific neurons that show aftereffects is similar across different stimulus types. K) Z-scored images show after-effect producing neurons that continued to fire for up to 5 min following stimulus removal. L) The proportion of aftereffects found in event-specific and panresponsive neurons decreases slightly on the second day of recording.

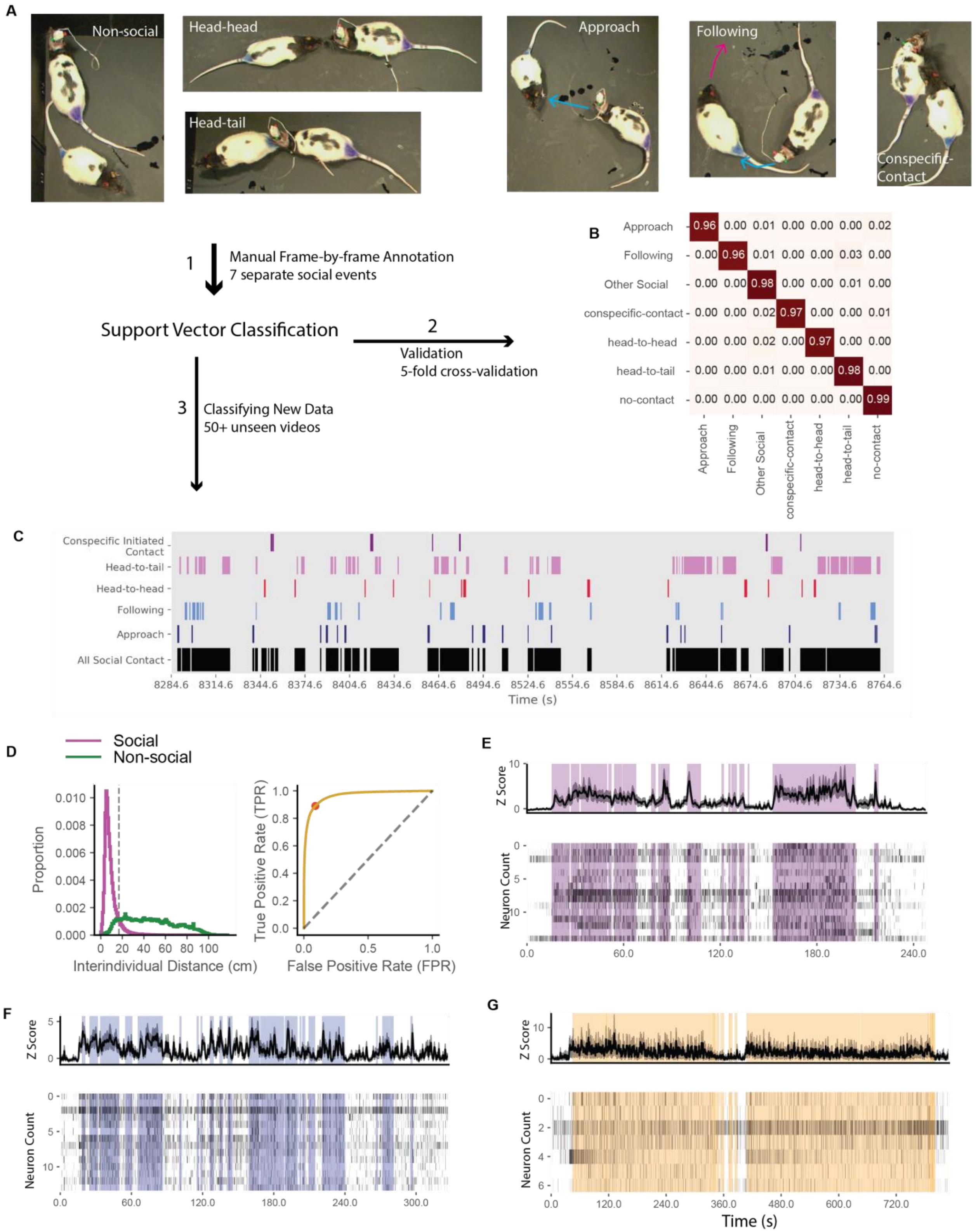

Fig. S7. Automatic Classification of Social Behavior. Related to Figure3.

A support vector classification was used to standardize the analysis of social behavior across all social interactions. A) 7 social interaction events (4 male-female events and 3 male-male

events) were manually annotated frame-by-frame for one of 7 distinct behaviors (see methods for description of the behaviors). Pictured are non-social behavior and 5 distinct social behaviors. B) We trained the SVC classifier on the manually annotated behavior using the features extracted from the automated tracking. The SVC classifier was validated using 5-fold cross validation, which yielded balanced accuracies of 0.97. (see methods for a full-description of the analysis). C) We used the trained SVC classifier to automatically classify social behavior in previously unseen videos. Using this classification method, we identified two layers of behavioral activity from each social event. #1 – whether the animals were interacting (social) or not interacting (non-social) and #2 – the timing of 5 distinct social behaviors (sensory behavior – i.e. head-head or head-tail, movement behavior i.e. approach or following and passive i.e. conspecific contact). D) To automatically calculate stimulus-interaction in nonsocial events, we calculated the optimal interindividual distance threshold from all social trials using ROC analysis (see methods, threshold = 16.8cm). This threshold was also applied to non-social trials to identify behavioral interactions with the stimulus. E-G) Interaction-modulated neurons across different stimulus presentations illustrate the presence of single-cell and neuronal populations that reliably track engagement in both social and non-social stimuli E, toy; F, males; G, food.

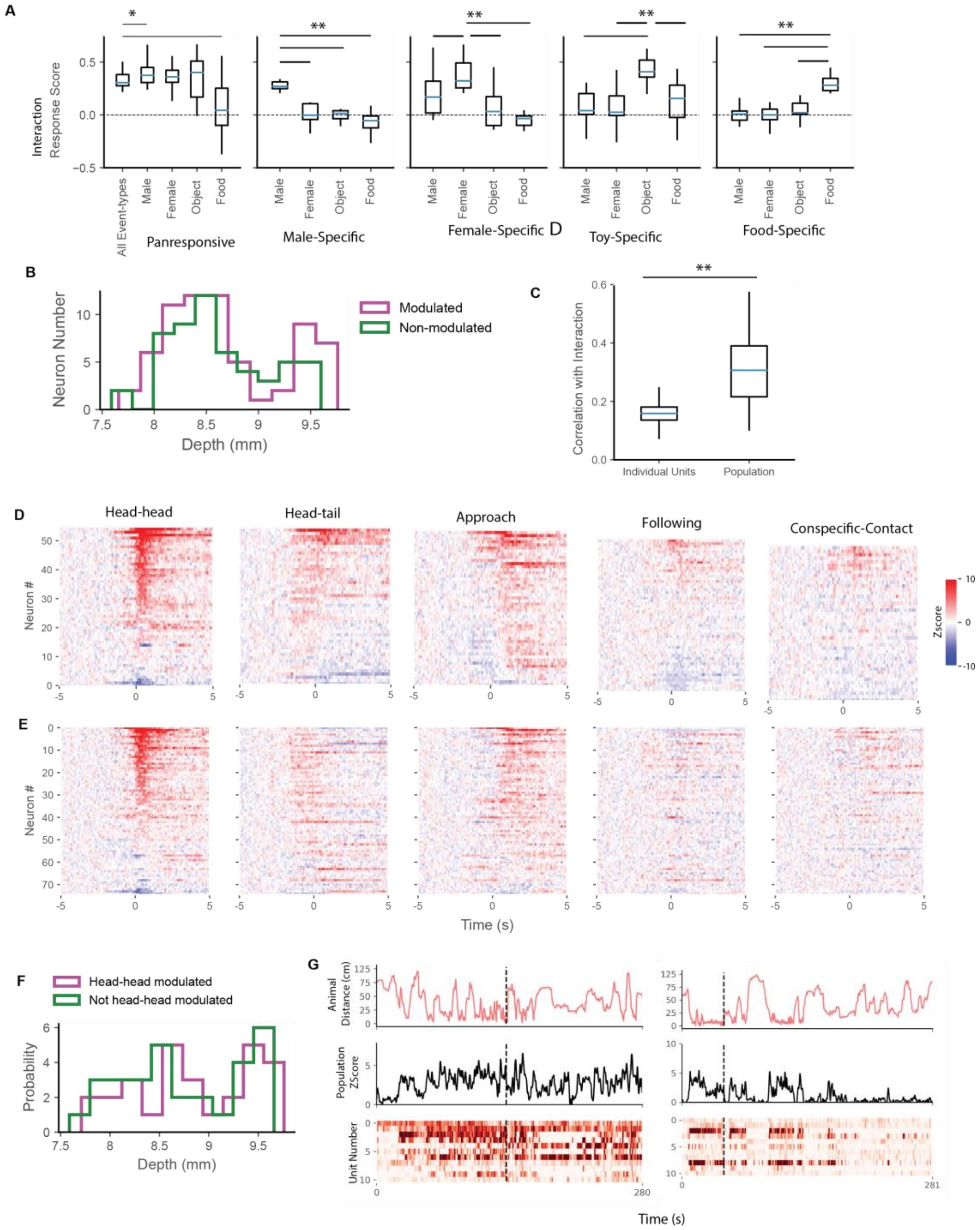

Fig. S8. Properties of neurons modulated by interaction with stimulus. Related to Figure3.

A) Interaction-modulated event-specific neurons only show modulation by the preferred type of stimuli. For example, male-specific neurons do not show the same levels of modulation to female, toy or food stimuli. (**p < 0.01, N = 8, 13, 15, 11 for male, female, toy and food exclusive engagement-modulated neurons, repeated-measures 1-way ANOVA). Panresponsive neurons show mixed selectivity (*p < 0.05, N = 15). B) Within responsive neurons, interaction-modulated neurons are not anatomically clustered compared to non-interaction modulated neurons. C) The population vector of interaction-modulated neurons is more highly correlated

with behavior compared to the average individual units per event (** $p < 0.01$, N = 58 events, unpaired Student's t-test). D) A proportion of neurons show responses to specific social behaviors (each heatmap is sorted according to maximal response). More units respond to head-head, head-tail contact and approach than to following and conspecific-contact. E) Some units show selectivity to multiple social behaviors (all heatmaps sorted by the individual neuron's response to head-head contact). F) Head-head modulated neurons are not anatomically localized any more than non head-head modulated neurons. G) Responsive units during interaction with a female conspecific do not show strong modulation as a result of mating (black dashed line) in these two example encounters. While activity appears to transiently dip after mating, this can be explained by the end of active interaction with the female conspecific as illustrated by the increase in animal distance immediately after mating.

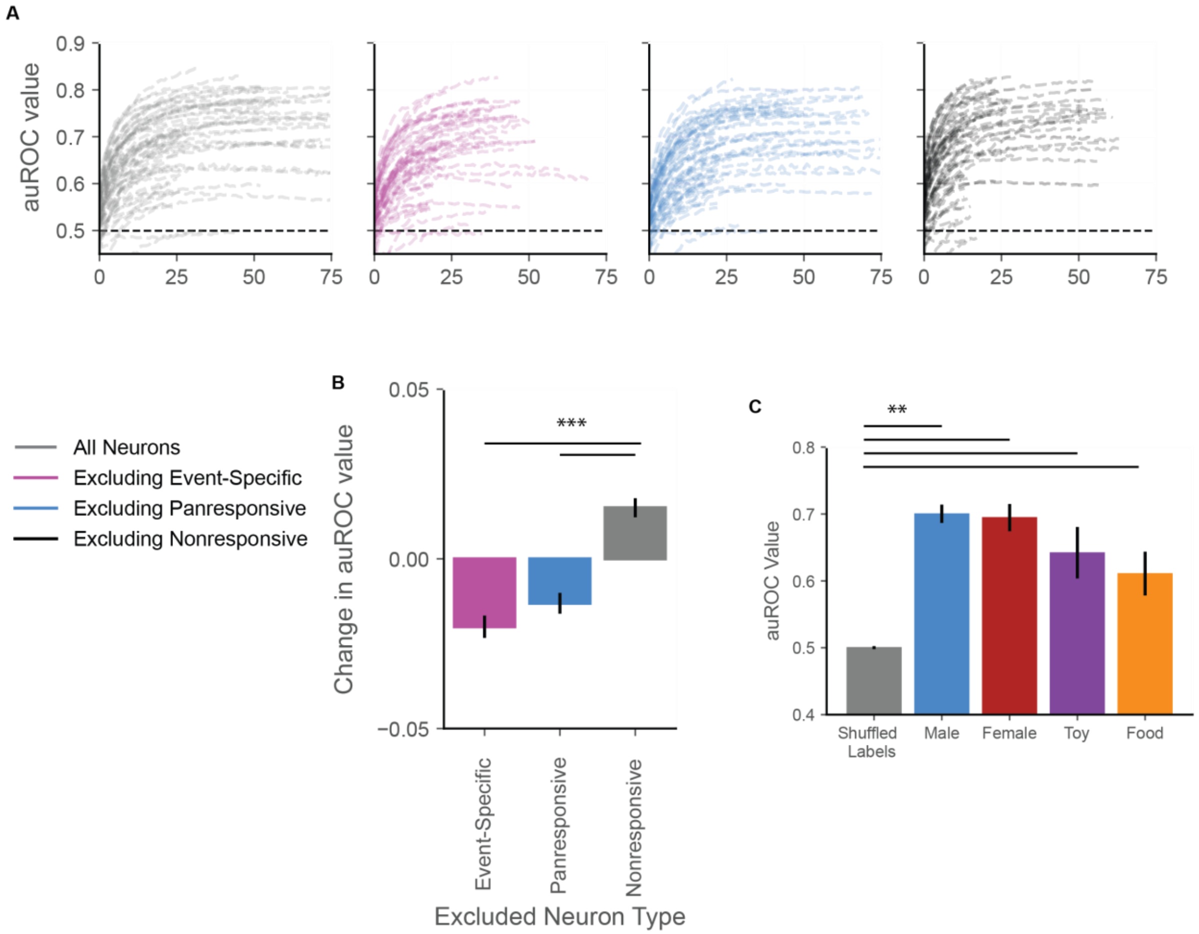

Fig. S9. Decoding interaction with stimuli from neural activity. Related to Figure3.
A) Accurate decoding of behavioral interaction with stimuli is highly dependent on the number of neurons used (each line represents a single event). Removing specific neuron types (i.e. event-specific, panresponsive or nonresponsive) mildly impacts the performance of the decoder. This is quantified in (B, **p< 0.01, event-specific: -0.02 ± 0.003, panresponsive: -0.01 ± 0.003, nonresponsive: 0.02 ± 0.003; mean ± SEM; N = 60 events, repeated-measures 1-way ANOVA). C) Behavioral interaction can be accurately decoded from event types with any of the 4 stimuli (**p<0.01, shuffled(60): 0.50 ± 0.003, male(27): 0.70 ± 0.01, female(17): 0.69 ± 0.02, toy(8): 0.64 ± 0.04, food(8): 0.61 ± 0.03; event-type(N): mean ± SEM; Kruskal-Wallis test)

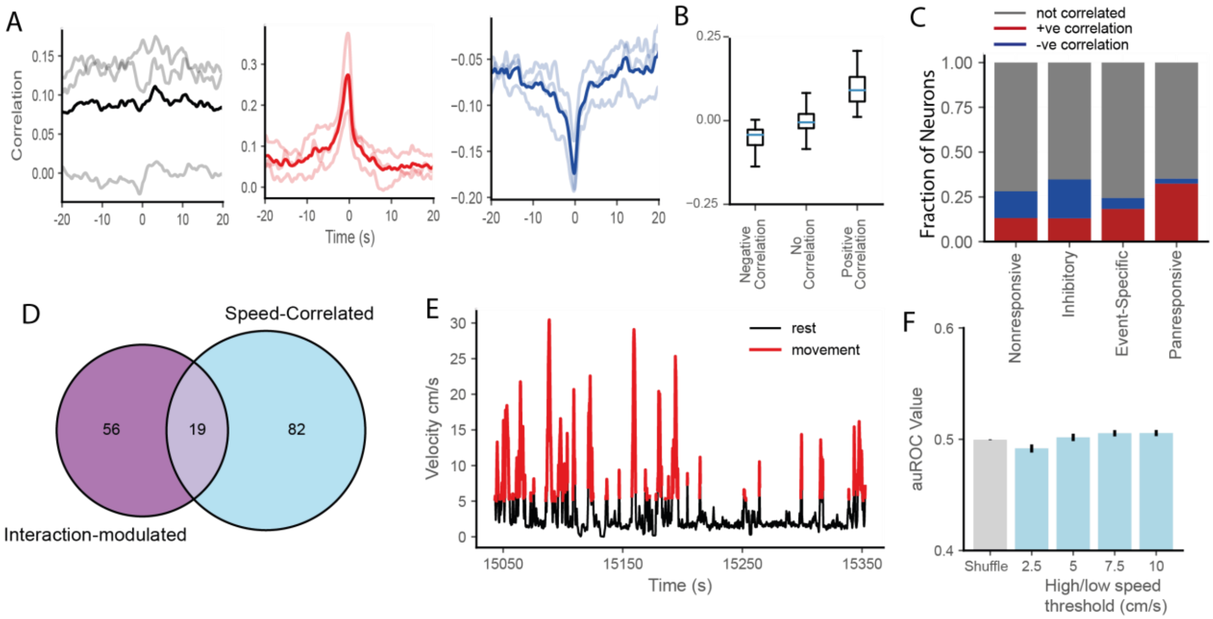

Fig. S10 BLA neurons show some correlation with speed but cannot decode movement. Related to Figure3.

A) Representative examples of BLA neurons either not correlated with speed (left), positively correlated (middle) or negatively correlated (right). The crosscorrelation of each unit with animal speed was calculated for baseline, event and post-event periods (lighter traces) and that average (darker trace) was used to determine the speed correlation. B) As a population, BLA units are only modestly correlated or anti-correlated with speed (negative: -0.04, none: -0.01, positive: +0.09, median correlation). C) Speed-correlated units are not over-represented by any one category of BLA neuron, however panresponsive units are more likely to be positively correlated with speed. D) Overall, there is little overlap between interaction-modulated units (see Figure3) and speed -correlated units. E) To test whether BLA firing can decode movement, behavioral data was divided into periods of rest or movement and tested using LDA decoding. F) Regardless of the exact threshold for rest versus movement, BLA firing activity was unable to decode movement. (p=0.09-0.92, high/low speed threshold categories compared to shuffle, N=60 events; repeated measures 1-way ANOVA)

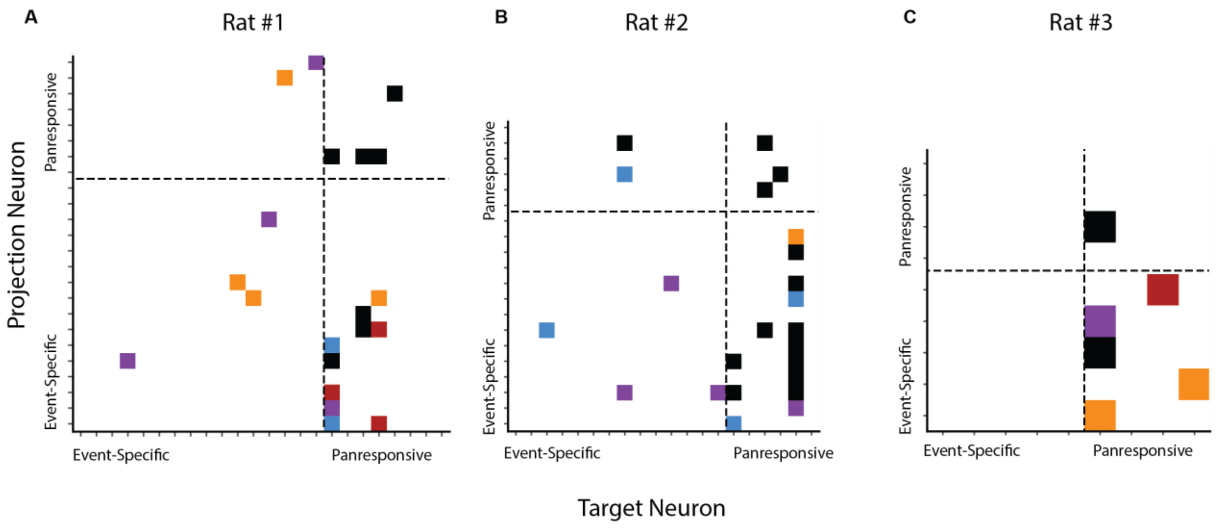

Fig. S11. Connectivity between event-specific and panresponsive neurons within individual rats. Related to Figure4.

A-C) Cross-correlated pairs of neurons within 3 separate rats during the first presentation of each stimulus. Colors represent whether the neuronal pair fired exclusively during a single presentation (red, blue, purple, orange represents female, male, toy, food respectively) or if the neuronal pair was active across multiple stimulus presentations (black, 2+ stimulus presentations). During the stimulus presentation the flow of neuronal information typically goes from event-specific to panresponsive neurons.
