# Supplementary material for: Representation of ethological events by basolateral amygdala neurons
Source: Cell Rep. 2022 Jun 7;39(10):110921. doi: 10.1016/j.celrep.2022.110921 (PMC9638002; doi:10.1016/j.celrep.2022.110921)
Supplement: Document S2. Article plus supplemental information [file mmc7.pdf]

Cell Reports

Report

Representation of ethological events by basolateral amygdala neurons

Graphical abstract

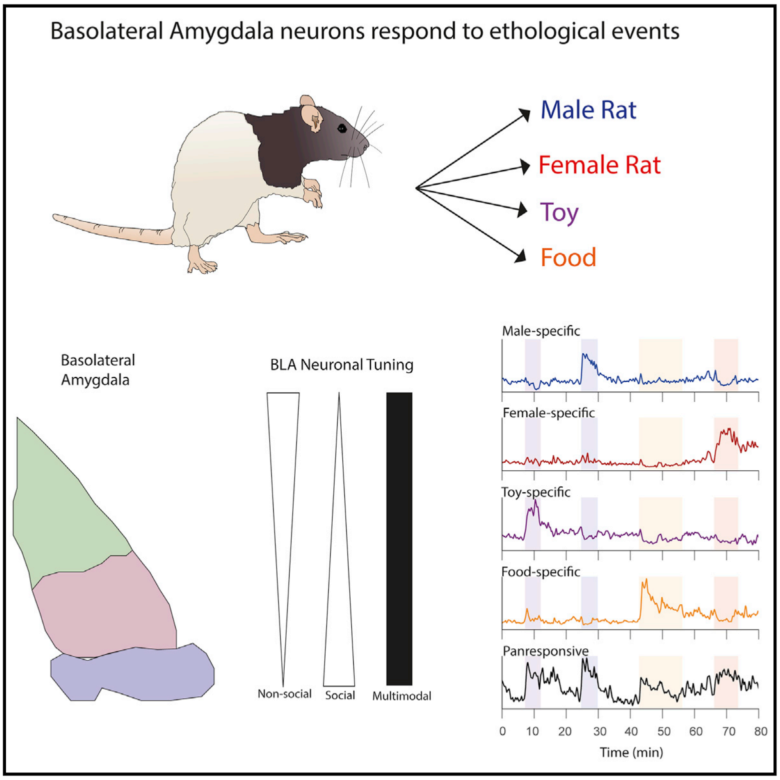

Highlights

- Basolateral amygdala (BLA) neurons respond selectively to salient stimuli
- After activation, BLA neurons can be modulated by the behavioral microstructure
- Firing persists in some BLA neurons long after the removal of the eliciting stimulus
- In the BLA micro-circuit, information flowed from more tuned to less tuned neurons

Authors

Cristina Mazuski, John O'Keefe

Correspondence

c.mazuski@ucl.ac.uk

In brief

Mazuski and O'Keefe identify basolateral amygdala (BLA) neurons that respond selectively to ethological events with large increases in firing activity. These BLA neurons differ in anatomical spread, response onset, and neuronal connectivity, with a subset continuing to fire for minutes after the end of the eliciting event.

Mazuski & O'Keefe, 2022, Cell Reports *39*, 110921
June 7, 2022 © 2022 The Author(s).
https://doi.org/10.1016/j.celrep.2022.110921

CellPress

Cell Reports

CellPress
OPEN ACCESS

Report

Representation of ethological events by basolateral amygdala neurons

Cristina Mazuski[1,3,*] and John O'Keefe[1,2]
[1]Sainsbury Wellcome Centre for Neural Circuits and Behavior, University College London, London W1T4JG, UK
[2]Department of Cell and Developmental Biology, University College London, London WC1E 6BT, UK
[3]Lead contact
*Correspondence: c.mazuski@ucl.ac.uk
https://doi.org/10.1016/j.celrep.2022.110921

SUMMARY

The accurate interpretation of ethologically relevant stimuli is crucial for survival. While basolateral amygdala (BLA) neuronal responses during fear conditioning are well studied, little is known about how BLA neurons respond during naturalistic events. We recorded from the rat BLA during interaction with ethological stimuli: male or female rats, a moving toy, and rice. Forty-two percent of the cells reliably respond to at least one stimulus, with over half of these exclusively identifying one of the four stimulus classes. In addition to activation during interaction with their preferred stimulus, these cells signal micro-behavioral interactions like social contact. After stimulus removal, firing activity persists in 30% of responsive cells for several minutes. At the micro-circuit level, information flows from highly tuned event-specific neurons to less specific neurons, and connection strength increases after the event. We propose that individual BLA neurons identify specific ethological events, with event-specific neurons driving circuit-wide activity during and after salient events.

INTRODUCTION

The basolateral amygdala has been assigned many different roles, including fear conditioning (Ledoux, 2000), valence association (Paton et al., 2006; Beyeler et al., 2016), exploratory behavior (Botta et al., 2020), and internal state (Gründemann et al., 2019). Single BLA neurons have largely been studied in the context of trained task-related activity, displaying responses to various cues, including neutral sensory information (e.g., auditory, tactile, olfactory) (Ono et al., 1995), fear and pain cues (Wolff et al., 2014; Corder et al., 2019), food (Liu et al., 2018), and conditioned valence responses (Beyeler et al., 2016). The presence of these small responses in broad, overlapping populations of BLA neurons has led to the suggestion that the complex, multisensory characteristics of naturalistic stimuli are likely to be encoded at the BLA circuit level as an aggregation of these low-amplitude noisy single-cell responses (Gründemann and Lüthi, 2015; Kyriazi et al., 2018; Kyriazi et al., 2020). In contrast, an earlier view proposed that single units could selectively identify events of significance to the animal (O'Keefe and Bouma, 1969). To distinguish between these alternative hypotheses, we recorded from large populations of neurons across the BLA using Neuropixels probes during several salient ethological events.

We recorded from 426 neurons throughout the basolateral complex (Figure 1A) of five male rats using single-shank Neuropixels probes. Each session consisted of a series of events (Figures 1B, 1C, and S1; Videos S1, S2, S3, and S4) during which the implanted rat could freely interact with an ethological stimulus placed in the recording chamber. Electrophysiological and video recording was continuous during a series of 5-min interactions with several males, females, a toy, or sweetened rice food with 5-min baseline recordings before and after each event. We classified different cell types on the basis of their waveforms, sensory/behavioral correlates, and location within the basolateral complex, and will discuss the involvement of each of these cell groups in different aspects of the events.

RESULTS

Single BLA cells identify the ethological event

The most striking result was that 23.5% of the cells showed a strong specific excitatory response to only one of the four classes of ethological stimuli (Figures 1 and S2 and STAR Methods). Figure 1C shows typical examples of these event-specific cells and quantifies the proportion of cells responding to each event (Figures 1D and S2). Typically, event-specific cells had a very low resting firing rate (median, 0.5 spikes s^{-1}) which increased many fold (3–6× on average) during the triggering ethological event. Importantly, the event-specific cells were highly selective, responding to only one class of event (for example, to all females or males) but showing virtually no response to other events (Figures 1C, 1D, and S2). We found no evidence that the BLA cells could discriminate among the different females or among the different males but cannot rule out the possibility that they might do so with further experience.

In addition to the event-specific responses, another 11.3% of the cells were multimodal, responding to more than one ethological event. Most of these neurons were panresponsive, responding to both social and non-social events (64% of panresponsive

This is an open access article under the CC BY license (http://creativecommons.org/licenses/by/4.0/).

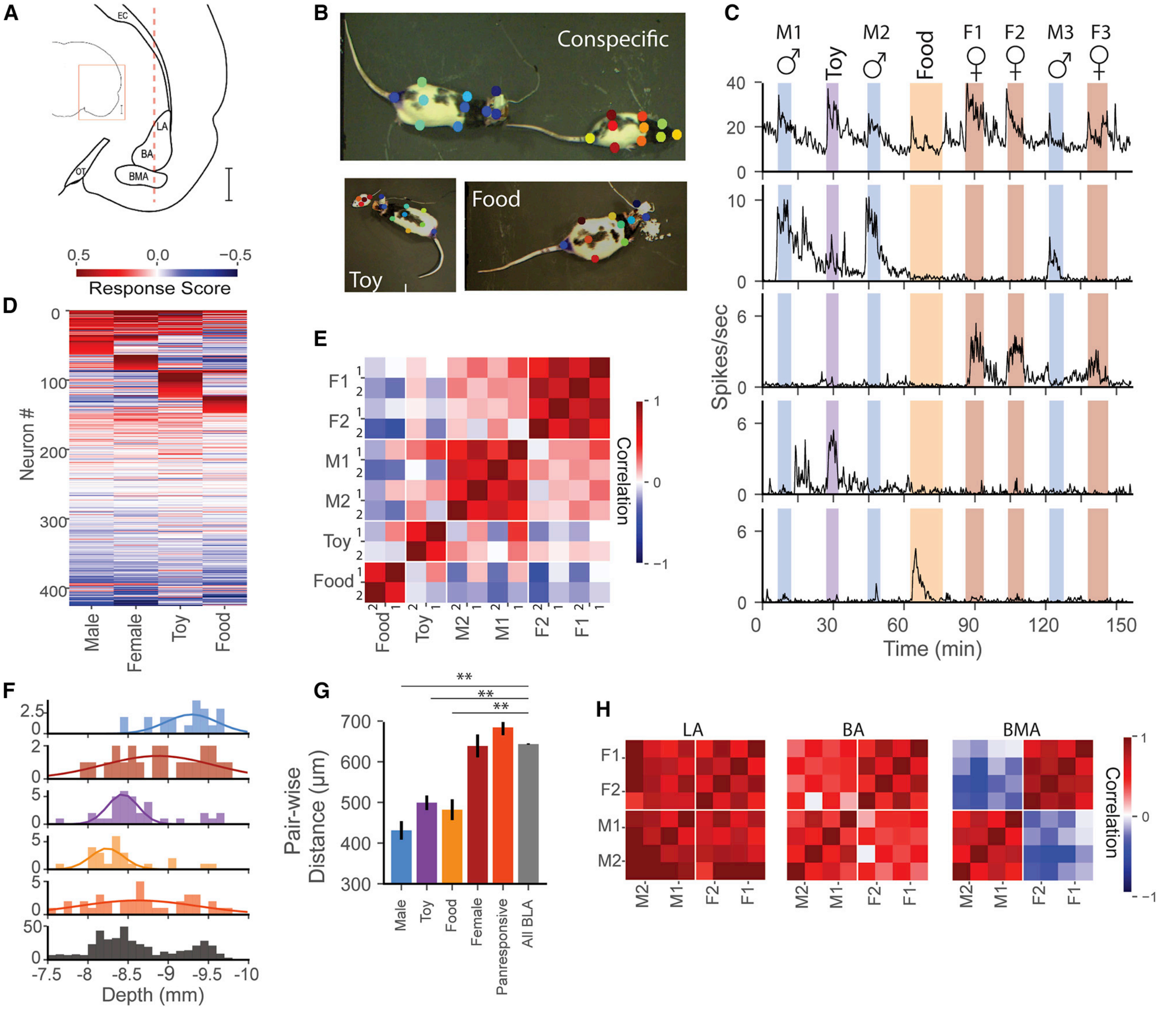

Figure 1. BLA neurons respond to salient social and non-social stimuli with regional differences

(A) Schematic of a coronal rat brain section depicting a Neuropixels probe (dashed red line) implanted in the BLA.

(B) The implanted rat freely engaged with different stimuli (top, social interaction with a conspecific; lower left, with a mobile toy mouse; lower right, eating sweet rice). Events were tracked using a combination of color marking and Deeplabcut (see Videos S1, S2, S3, and S4; Figure S1; and STAR Methods).

(C) Each recording session consisted of 5-min event blocks of free interaction with one of four classes of stimuli interspersed with 5-min control periods. Stimuli consisted of (top) up to three different males (♂), up to three females (♀), a toy mouse (toy), and sweetened rice (food). (Below) Examples of diverse responses in subsets of BLA single neurons: from top to bottom, panresponsive, male-specific, female-specific, toy-specific, and food specific.

(D) Average response score for all BLA single units ordered by response strength and category. From top to bottom, panresponsive, social (male specific, female specific), non-social (toy specific, food specific, no responses, and decreased firing).

(E) Population correlation of response score vectors from all responsive units across individual stimulus presentations shows strong correlations within events of the same type and weaker correlations across events of different types. Rows and columns represent the first (1) and second (2) presentations of a given event type.

(F and G) (F) Male, object and food event-specific neurons are clustered along the dorsal-ventral axis with (G) lower pairwise distances than the average neuron. Female-specific and panresponsive neurons are not clustered. $^{**}p < 0.01$. Male: 431.2 ± 22.74 (20). Toy: 499.1 ± 17.85 (34). Food: 481.9 ± 25.52 (24). Female: 638.5 ± 28.1 (22). Panresponsive: 684.1 ± 18.91 (36). All BLA: 643.3 ± 1.60 (426). Neuron-type (N): micrometer distance between neuron pairs. Mean ± SEM; Kruskal-Wallis test.

(H) Population response vector correlation shows little discrimination between male and female conspecifics in LA, and greater discrimination in BA and BMA.

neurons responded to three or more events), and the majority of these had narrower waveforms and higher baseline firing rates compared with the more silent putative pyramidal neurons (Figure S2). A smaller proportion of neurons that were not classified as panresponsive or event specific instead decreased in firing to one or more stimuli (7.7%). There were no significant differences

in response intensity to different events, suggesting that, as a whole, the BLA does not prefer one type of ethological stimulus over another (Figure S2).

We observed strong population correlations between presentations of the same event type, despite significant behavioral variability during the event period (Figures 1E and S1). The selectivity of these responses for stimulus class was so strong that it was possible to use a decoder to identify the stimulus with 77% accuracy (Figure S3). Decoding of stimulus identity was possible with relatively few neurons (Figure S3, performance plateaus with 10 neurons) and that effect was largely, but not exclusively, driven by the contribution of event-specific neurons (Figure S3). In a subset of neurons reliably tracked across 2 days of recording, single-unit responses to events remained stable and specific (Figure S4).

Different stimuli are represented in different subdivisions of the BLA

The large number of electrodes and their density along the Neuropixels probes, together with careful placement of the probes within the BLA, allowed simultaneous recording from two or more subdivisions of the BLA including lateral (LA), basal (BA), and basomedial (BMA) in each animal (Figure S5; STAR Methods). All three subdivisions contained roughly the same percentage of multimodal panresponsive cells (11%–12%) and the same percentage of cells that decreased firing (7%–8%) but differed in number and type of event-specific neurons. LA contained fewer responsive neurons, fewer putative interneurons, and had lower average baseline firing rates compared with the BA and BMA (Figures S2 and S5). Non-social (food and toy) events were more highly represented in the LA and BA (77% and 67% of event-specific neurons), while social events were more prominent in the BMA (74%) (Figures 1F, 1G, and S5). At the population level, this resulted in BMA and, to a slightly lesser extent, BA distinguishing strongly between males and females (Figure 1H). Decoding of social stimuli improved at greater depths corresponding to the ventral BA and BMA (Figure S5).

Increased firing in neurons temporally linked to identification of the ethological event

Responsive BLA units exhibited large sudden increases in firing activity around the time of event onset (Figures 2A and 2B). To further understand the temporal dynamics of this increase, we divided the event start into distinct periods (presentation, interaction, and direct contact; Figure 2C; STAR Methods). The duration of the event start epoch varied based on the behavior of the implanted rat and the stimulus (Figure 2D). Using this intrinsic variability, we observed that panresponsive cells showed a brief transient period of activity at both the presentation and removal of the stimulus regardless of the stimulus identity (Figures 2E, 2F, and S6), while the largest increase in firing in both event-specific and panresponsive neurons occurs seconds prior to direct contact with the stimulus. This increased activity likely reflects awareness of stimulus identity or the decision to interact (Figures 2G–2I) and was not linked to any identifiable behavior. Unit activity profiles showed similar temporal patterns across the four classes of stimuli despite their very different sensory natures and across 2 days of recording (Figure S6), strongly suggesting identification of the event and not its specific sensory characteristics.

Firing persists in a subset of BLA neurons after stimulus removal

In contrast to the sharp increases in firing activity seen at event onset, elevated firing continued in approximately 30% of responsive neurons after the removal of the stimulus (33% of event-specific neurons and 27% panresponsive compared with 9% nonresponsive; Figures 2J and 2K). Figure 2A shows examples of this after-response in a male-responsive cell (top) and a panresponsive cell (bottom; also Figure 1C, top two panels). The period of persistence differed between cells and even within cells during different presentations of the same event, with some elevated firings lasting the entire post-event and others decaying at a faster rate. All stimuli were capable of producing these aftereffects and aftereffects were present on the second day of recording in slightly fewer neurons (Figure S6). Compared with baseline, the population firing activity in the post-event period was capable of decoding stimulus identity, suggesting that this activity may function as a memory trace (Figures 2L and 2M).

Firing rates of activated neurons are modulated by specific social interactions

Once firing activity was triggered by an event, behavioral interaction caused short-term fluctuations in firing rates (Figures 3A–3C). To study these micro-behaviors, we classified key social behaviors using an support vector machine classifier (SVC) trained on manually annotated video data (Figure S7 and Video S5). From the resulting automatically classified social behavior, we calculated the inter-individual distance that best discriminated social versus non-social interaction and applied this metric to non-social events (i.e., toy and food; Figure S7; see STAR Methods) as well. Event-specific neurons only showed micro-behavioral modulation in response to their own tuned stimulus (Figures S7 and S8). The micro-behavioral correlation was higher at the population level than at the single-unit level, and there was no difference in anatomical location between units that showed micro-behavioral modulation and those that did not (Figure S8). Linear discriminant analysis (LDA) decoding on BLA population activity allowed reliable decoding of micro-behavioral modulation in all four event types (Figures 3D and S9), and decoding performance was strongly dependent on the number of neurons used (Figure S9). We further examined social events to see whether single units fired in response to common social micro-behaviors, including sensory behaviors (head-to-head contact, head-to-tail contact), movement-related behaviors (approach and following), and passive contact initiated by the conspecific. We found units showing increases or decreases in response to all these specific social behaviors, with head-to-head contact particularly well represented (Figures 3E–3I and S8). Although these neurons reliably responded to specific behaviors, their responses were not unique to these behaviors (Figure S8). Less common social behaviors, such as mating between the implanted rat and a female conspecific, were not obviously reflected in BLA neuronal activity (Figure S8).

To examine the influence of motor activity on BLA neurons, we cross-correlated firing activity with the speed before, during,

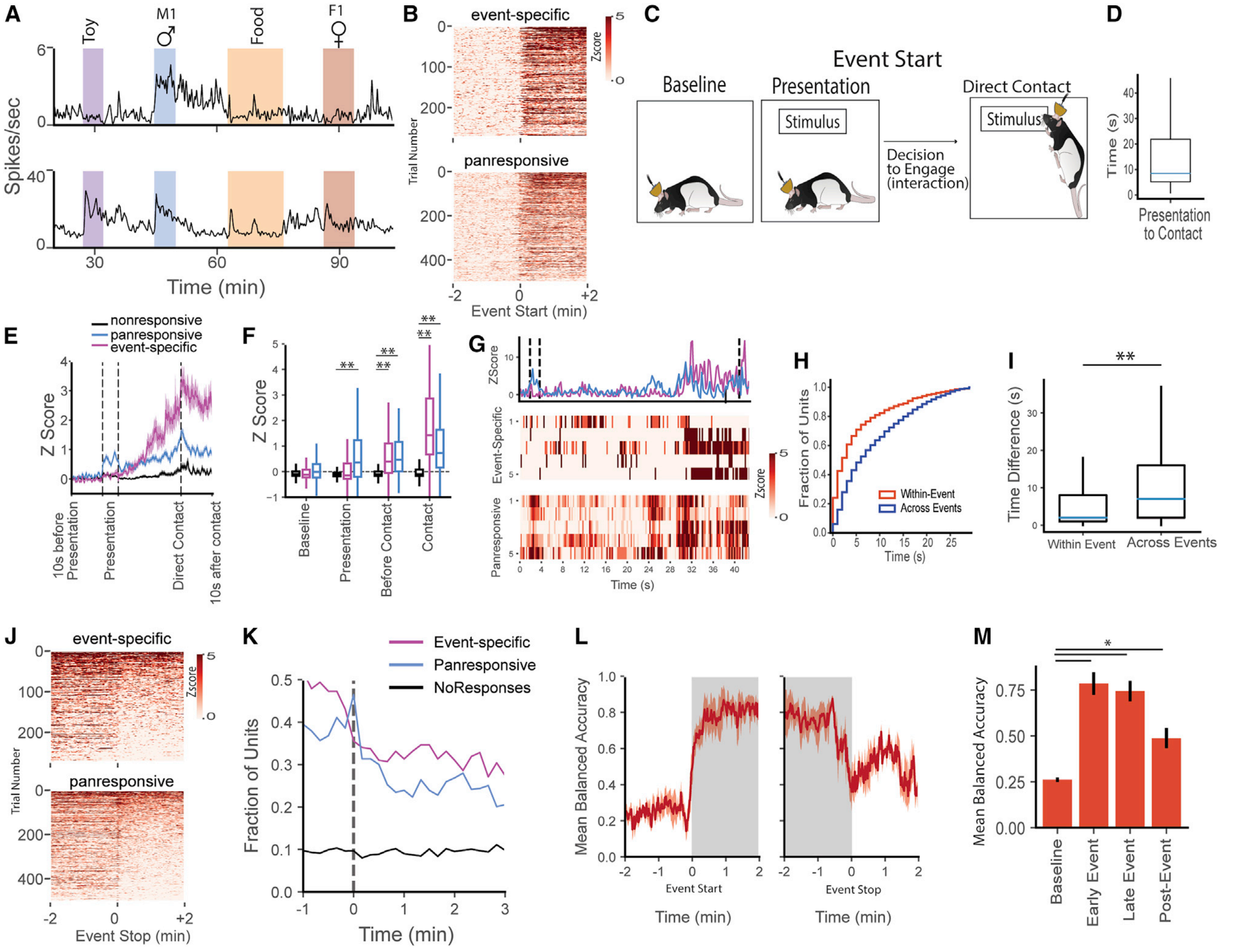

Figure 2. BLA neurons fire at the beginning of, during, and after an ethological event

(A) Abrupt increase in activity at event onset and its slow decay afterward in typical event-specific (top) and panresponsive (bottom) neurons.

(B) Event-specific and panresponsive neurons show this pattern of sharp increase in activity with sustained activity for several minutes following event start across different events.

(C) Event start can be further divided into distinct periods based on behavior. After baseline recording, the stimulus is placed at the opposite end of the chamber (presentation). Following a decision to engage, there is direct physical contact between the implanted rat and the stimulus.

(D) There is a large variation in time to contact after presentation, with an average of about 10s.

(E andF) (E) Warping each event start into a common time frame reveals differences between panresponsive and event-specific neurons, with increased activity occurring during the presentation period exclusively in panresponsive neurons and event-related firing starting in both event-specific and panresponsive before contact. This effect is quantified in (F). $^{**}p < 0.001$; significant effects for neuron-type, time point, and interaction; N = nonresponsive (2,960), panresponsive (320), event-specific (423) trials; ordinary two-way ANOVA.

(G) Step-like increases in population activity prior to direct contact in (E) are caused by clusters of neurons rapidly switching on within a single event, as seen in this representative example.

(H and I) Within a single-event trial, individual neurons initiate their firing within seconds of each other, indicating a uniform population response to the eliciting event. This is quantified by comparing the relative timing of firing activity of responsive neurons within individual events with the relative timing of firing activity of the same neurons across different events (see STAR Methods). $^{**}p < 0.01$; N = within-event (3,231), across-event(2,026) neuron pairs; Kolmogorov-Smirnov test.

(J) In contrast to the rapid onset of activity at event start, after event offset, many neurons continue to show elevated firing activity for several minutes (aftereffects).

(K) This elevated activity occurs in approximately 30% of event-specific and panresponsive neurons and persists long after event stop.

(L) Stimulus identity can be reliably decoded from neuronal activity both during the event (area shaded gray) and after the stimulus is removed. Decoding accuracy is significantly higher than baseline (M), ($^{*}p < 0.05$; baseline, 0.26 ± 0.01; early event, 0.78 ± 0.06; late event, 0.74 ± 0.06; post event, 0.48 ± 0.05; mean ± SEM; N = 4 rats; repeated measures one-way ANOVA). Data on box plots correspond to the median and 25th–75th percentiles.

and after events and identified 28% of BLA neurons that show some speed correlation. These speed-correlated neurons only modestly overlapped with BLA neurons encoding event micro-behaviors (Figures 3B and 3C), and firing activity was unable to decode whether the animal was moving or stationary (Figure S10).

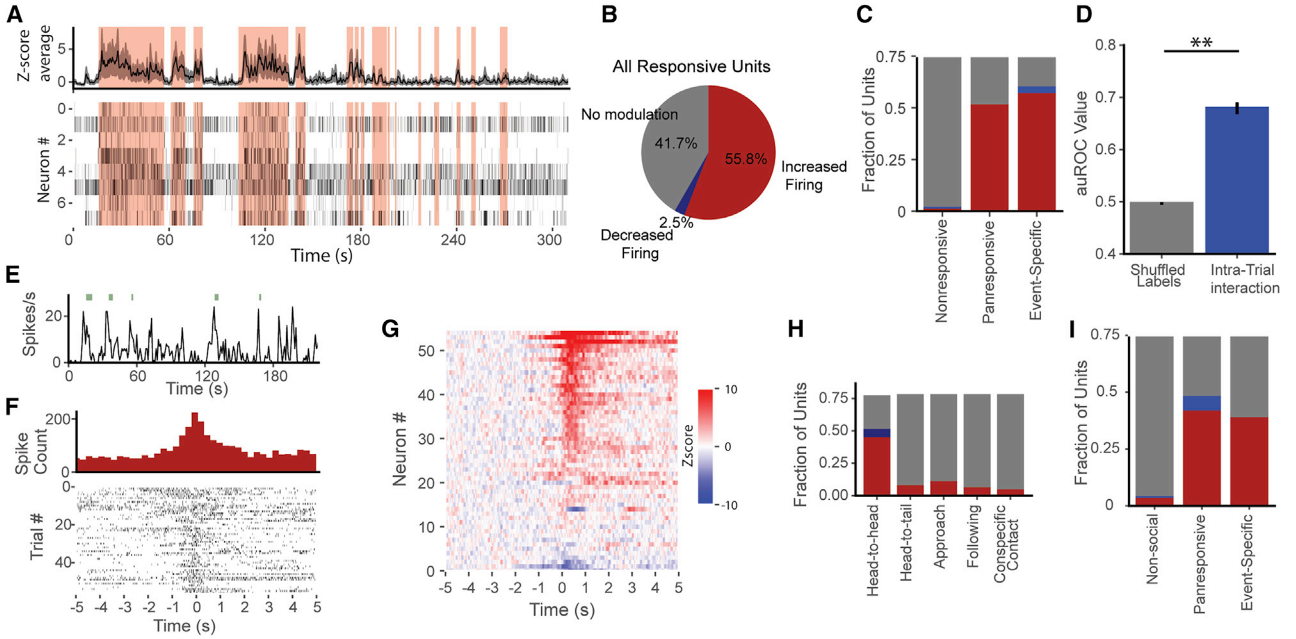

Figure 3. BLA neuronal activity is modulated by interaction with the stimulus with a subset of neurons reacting to discrete social behaviors

(A) Representative trial showing modulation of firing activity (population, top; single-unit raster plots bottom) by interaction with a female conspecific (social behavior shaded in pink).

(B and C) (B) The majority of both event-specific and panresponsive BLA units are modulated by social interaction in the same proportion (C).

(D) Behavioral interaction with a stimulus can be decoded from neuronal activity (**$p < 0.01$; shuffled, 0.50 ± 0.002; actual, 0.68 ± 0.01; mean $\pm$ SEM; $N = 60$ events; paired Student's t test).

(E) Representative example of a single unit showing time-locked firing increases during head-to-head contact (green ticks). Note that the unit also bursts outside these contacts.

(F) Peri-event histogram of the unit in (E) showing all instances of head-to-head contact (at 0).

(G) *Z* scored peristimulus time histogram (PSTH) traces for all head-to-head contact units sorted by magnitude of response.

(H and I) (H) The proportion of units that increase (red) and decrease (blue) firing to specific social behaviors by behavior type and (I) neuronal class.

Event-specific neurons drive firing in panresponsive neurons

We identified the directionality of firing and connection strength from significantly cross-correlated BLA neurons throughout the entire recording session (STAR Methods; Figures 4A, 4H–4J). This revealed a clear circuit structure where, typically, several event-specific neurons conveyed information to single panresponsive neurons through putative monosynaptic inputs (Figures 4B–4F and S11). Connected neurons tended to lie within 200 μm of each other (Figure 4G). To test how these connections changed in response to presentation of the ethologically relevant stimulus, we calculated the connection strength from correlated neurons before, during, and after the first presentation of each stimulus type (Figures 4K–4M). Connection strength significantly increased during the post-event period both at the population level and within continuously active pairs of correlated neurons (Figures 4N and 4O). These results support a model where firing from event-specific neurons drives activity in panresponsive neurons, likely driving increased plasticity within the BLA.

DISCUSSION

The BLA contains information about many important aspects of the ethological events studied in this experiment. Unlike other areas often described as part of the social circuitry (e.g., prefrontal cortex, medial amygdala) (Li et al., 2017; Levy et al., 2019), the BLA as a whole did not show a strong preference for social or non-social events. Instead, such a preference was manifested at the level of BLA subdivisions with non-social (food and toy) events represented in the LA and BA and social conspecific events in the ventral BA and BMA. The BMA and, to a slightly lesser extent, BA further distinguished strongly between males and females. Subnuclear preferences for different events are consistent with reported anatomical differences in BLA connectivity (Beyeler et al., 2018). However, female conspecifics activated event-specific neurons across the whole BLA, which may be due to female stimuli recruiting multiple downstream circuits compared with male stimuli; for example, circuits necessary for identifying and responding to sexual receptivity. The similarity in temporal response profiles across events and anatomical subdivisions suggests that, despite the recruitment of different neuronal clusters, the coding principles used by BLA neurons during different ethological events are likely similar at the cellular and circuit level.

At the single-cell level, we identified two main classes of BLA neurons. Approximately 30% of BLA neurons (event-specific neurons) exhibited well-tuned macro-scale increases in firing to one of the four ethological events in our study. These neurons

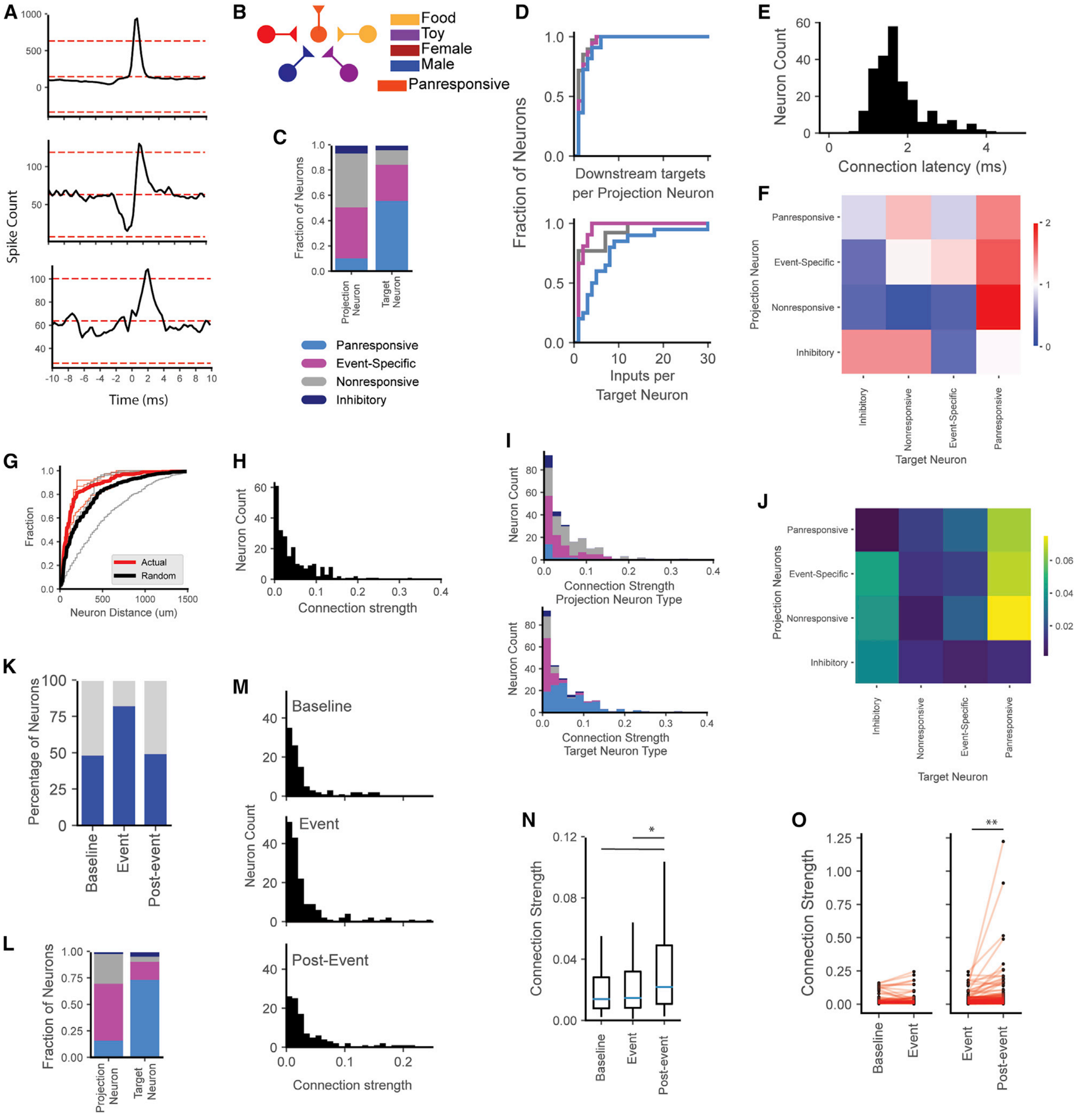

Figure 4. Event-specific neurons drive firing activity in panresponsive neurons

(A) BLA cells are highly interconnected. Representative cross-correlograms show short-latency, putative monosynaptic connectivity between BLA neurons. Connected target neurons exhibited a sharp increase in firing within milliseconds of the projection neuron firing (see STAR Methods).

(B) BLA circuit model suggested by the cross-correlation results where different event-specific neurons drive firing in panresponsive neurons.

(C) Throughout the entire recording session, we identified 234 pairs of BLA neurons with short-latency cross-correlations. Within these pairings, the majority of projection neurons were event specific or nonresponsive, while the majority of target neurons were panresponsive.

(D) Individual projection neurons had few downstream targets (top), while target neurons, specifically panresponsive neurons, often received input from many projection neurons.

(E) The majority of correlated neuron pairs fired within 1–2 ms of each other, suggesting putative monosynaptic connections.

(F) Connectivity between specific types of neurons was seen more often in the BLA than expected by chance (>1 = more likely than chance, <1 = less likely than chance).

(G) The majority of connected neuron pairs were located within 200 μm of each other, closer than expected by chance.

(legend continued on next page)

were anatomically localized, encoded the identity of the ethological stimuli, and continued to signal the most recent event for minutes after its termination. They drove firing in a second class of BLA cells (panresponsive neurons), which fired in response to multiple ethological events, signaled the beginning and the end of each event by brief bursts of action potentials, and received input from multiple neurons, including event-specific neurons and other nonresponsive neurons.

Given the stable responses of individual event-specific neurons to a single ethological stimulus, we suggest that these BLA neurons may be hard-wired to respond to innate naturalistic stimuli (for example, male or female conspecifics, food, or prey), and hypothesize that the detection of these stimuli is one of their primary functions, reminiscent of neuronal responses to salient stimuli like faces previously reported in other brain areas (Freiwald and Tsao, 2010). To our surprise, neuronal responses to the moving toy mouse were both selective and stable. Based on the rat's behavioral response to this stimulus (i.e., high levels of engagement, chasing, and occasional chewing), we suggest that the rat viewed the toy as the real thing (i.e., as prey). On this view, we would predict that many of the neurons in the BLA classified as nonresponsive would respond selectively to other ethologically significant stimuli, such as rat pups, nests, other salient locations, or predators. In our study, nonresponsive neurons resembled event-specific neurons with low baseline firing rates, broad waveforms, and similar directional connectivity to panresponsive neurons. We further speculate that continued experience with different males, females, or types of food might lead these cells to begin to distinguish individual animals or foods, respectively.

Once activated by a given event, the firing patterns of many BLA cells are modulated by the microstructure of the event, most notably by active interaction with the stimulus. This suggests that activated clusters of BLA neurons represent finer-grained details of the event, including specific sensory social behaviors, most predominantly head-to-head contact. Our study looked only at a limited set of specific behaviors associated with sensory inputs, and it is likely that BLA neurons are modulated by a host of sensory and behavioral factors (e.g., smells, taste, auditory vocalizations). For example, it is known that social transmission of olfactory information about edible foods is transmitted by the breath of the informing animal and is amygdala dependent (Wang et al., 2006). The firing modulation seen in our study is similar to previously described BLA state-dependent activity (Gründemann et al., 2019; Botta et al., 2020; Fustiñana et al., 2021).

In addition to providing evidence that the BLA is involved in identification of ethologically salient stimuli, the results show that single neurons continue to provide information about the previous event for minutes after its cessation. The BLA has a well-established role in memory consolidation and recall (McGaugh, 2004), and inhibition of the BLA or activation of specific engram cells can affect this process (Han et al., 2009; Liu et al., 2012). The aftereffects seen in our data might support short-term active memories allowing subsequent recognition and recall of the event. They might also be required for consolidation in other brain areas or the elicitation of long-term hormonal responses by these events. These effects have been observed at smaller scales in other brain regions (e.g., cortex, hypothalamus) (Curtis and Lee, 2010; Kennedy et al., 2020), but the BLA responses are unique in their long time scales, often exceeding 5 min in duration. We hypothesize that this long-term persistent BLA firing originates with the BLA event-specific neurons, which then trigger firing in BLA panresponsive neurons. These panresponsive neurons may release neuropeptides or other signaling molecules that maintain and reinforce high activity in the BLA for long periods of time. It remains to be seen how BLA projection neurons relate to the functional classes described in this study (e.g., do the same event-specific neurons that send local BLA connections to panresponsive neurons also project to other brain structures?).

Limitations of the study

In this study, we allowed rats to engage in a semi-naturalistic task where they could freely interact with four distinct stimuli types. While this design allowed us to directly compare the neuronal responses between these stimuli to begin to understand the BLA's neuronal heterogeneity, many questions remain. BLA population and single-cell activity often remained elevated for longer than the 10 min between stimulus presentations. Since firing from strongly activated event-specific neurons sometimes extended into additional event periods, a complete characterization of aftereffect time course was not possible. Future studies designed to explicitly measure aftereffects would benefit from fewer stimuli presented, longer recordings, and explicit control of behavior after stimulus removal to fully understand their role in BLA activity. On the other hand, to understand whether silent nonresponsive BLA neurons might be recruited by other ethological stimuli, we intend to present the rat with a greater range of stimuli, including sensory-specific stimuli (e.g., auditory tones, odors) and multisensory stimuli, as well as additional ethological ones.

STAR★METHODS

Detailed methods are provided in the online version of this paper and include the following:

- KEY RESOURCES TABLE
- RESOURCE AVAILABILITY

(H–J) (H) Connection strength between pairs of neurons (see STAR Methods). Panresponsive neurons were more likely to receive stronger input from event-specific, nonresponsive, and other panresponsive neurons (I and J).

(K) Number of neuron pairs with significant cross-correlations before, during, and after the first presentation of each stimulus.

(L) During these events, projection neurons were once again more likely to be event-specific or nonresponsive neurons, whereas target neurons were predominantly panresponsive.

(M and N) (M) Connection strength increases in the post-event period. This is quantified in (N). *$p < 0.05$, N = baseline (100), event (157), post-event (107) correlated neuron pairs; Kruskal-Wallis test.

(O) Increase in connection strength of individual neuron pairs between the event and post-event period (baseline to event connectivity: $p = 0.54$, N = 65 neuron pairs; event to post-event connectivity, **$p < 0.01$, N = 157 neuron pairs; Student's paired t test).

- Lead contact
- Materials availability
- Data and code availability

- EXPERIMENTAL MODEL AND SUBJECT DETAILS
  - Animals
- METHOD DETAILS
  - Neuropixels implantation
  - Histology
  - Data collection/experimental setup
  - Single-cell isolation from multi-unit recordings
  - Synchronization of video and electrophysiology
  - Tracking from video data
  - Classifying responsivity of individual neurons
  - Classification of putative pyramidal neurons and putative interneurons
  - Population correlation of response scores
  - Decoding stimulus identity from neural activity
  - Quantification of differences in firing onset and firing offset
  - Automatic classification of behavior from video data
  - Calculating unit response to behavior
  - Decoding behavior from neural activity
  - Cross-correlation analyses
  - Tracking neurons across two days of recording
  - Speed correlation
- QUANTIFICATION AND STATISTICAL ANALYSIS
  - Statistics

SUPPLEMENTAL INFORMATION

Supplemental information can be found online at https://doi.org/10.1016/j.celrep.2022.110921.

ACKNOWLEDGMENTS

We thank Dr. Yoh Isogai and Daniel Regester for the design of the retrievable Neuropixels holder and O'Keefe laboratory members for helpful discussions. This project has received funding from the European Union's Horizon 2020 research and innovation program under the Marie Sklodowska-Curie grant agreement no. 840562, the Sainsbury Wellcome Centre Core Grant from the Gatsby Charitable Foundation and Wellcome Trust (090843/F/09/Z), and Wellcome Trust Principal Research Fellowship (Wt203020/z/16/z).

AUTHOR CONTRIBUTIONS

Conceptualization, C.M. and J.O.; analysis/investigation, C.M.; funding: C.M. and J.O.; writing, C.M. and J.O.

DECLARATION OF INTERESTS

The authors declare no competing interests.

Received: December 27, 2021
Revised: April 1, 2022
Accepted: May 12, 2022
Published: June 7, 2022

REFERENCES

Beyeler, A., Namburi, P., Glober, G., Simonnet, C., Calhoon, G., Conyers, G., Luck, R., Wildes, C., and Tye, K. (2016). Divergent routing of positive and negative information from the amygdala during memory retrieval. Neuron *90*, 348–361. https://doi.org/10.1016/j.neuron.2016.03.004.

Beyeler, A., Chang, C.J., Silvestre, M., Leveque, C., Namburi, P., Wildes, C.P., and Tye, K.M. (2018). Organization of valence-encoding and projection-defined neurons in the basolateral amygdala. Cell Rep. *22*, 905–918. https://doi.org/10.1016/j.celrep.2017.12.097.

Botta, P., Fushiki, A., Vicente, A.M., Hammond, L.A., Mosberger, A.C., Gerfen, C.R., Peterka, D., and Costa, R.M. (2020). An amygdala circuit mediates experience-dependent momentary arrests during exploration. Cell *183*, 605–619.e22. https://doi.org/10.1016/j.cell.2020.09.023.

Corder, G., Ahanonu, B., Grewe, B.F., Wang, D., Schnitzer, M.J., and Scherrer, G. (2019). An amygdalar neural ensemble that encodes the unpleasantness of pain. Science *363*, 276–281. https://doi.org/10.1126/science.aap8586.

Curtis, C.E., and Lee, D. (2010). Beyond working memory: the role of persistent activity in decision making. Trends Cogn. Sci. *14*, 216–222. https://doi.org/10.1016/j.tics.2010.03.006.

Freiwald, W.A., and Tsao, D.Y. (2010). Functional compartmentalization and viewpoint generalization within the macaque face-processing system. Science *330*, 845–852. https://doi.org/10.1126/science.1194908.

Fustiñana, M.S., Eichlisberger, T., Bouwmeester, T., Bitterman, Y., and Luthi, A. (2021). State-dependent encoding of exploratory behaviour in the amygdala. Nature *592*, 267–271. https://doi.org/10.1038/s41586-021-03301-z.

Gründemann, J., Bitterman, Y., Lu, T., Krabbe, S., Grewe, B.F., Schnitzer, M.J., and Luthi, A. (2019). Amygdala ensembles encode behavioral states. Science *364*, eaav8736. https://doi.org/10.1126/science.aav8736.

Gründemann, J., and Lüthi, A. (2015). Ensemble coding in amygdala circuits for associative learning. Curr. Opin. Neurobiol. *35*, 200–206. https://doi.org/10.1016/j.conb.2015.10.005.

Han, J.H., Kushner, S.A., Yiu, A.P., Hsiang, H.L.L., Buch, T., Waisman, A., Bontempi, B., Neve, R.L., Frankland, P.W., and Josselyn, S.A. (2009). Selective erasure of a fear memory. Science *323*, 1492–1496. https://doi.org/10.1126/science.1164139.

Jun, J.J., Steinmetz, N.A., Siegle, J.H., Denman, D.J., Bauza, M., Barbarits, B., Lee, A.K., Anastassiou, C.A., Andrei, A., Aydin, C., et al. (2017). Fully integrated silicon probes for high-density recording of neural activity. Nature *551*, 232–236. https://doi.org/10.1038/nature24636.

Kennedy, A., Kunwar, P.S., Li, L., Stagkourakis, S., Wagenaar, D.A., and Anderson, D.J. (2020). Stimulus-specific hypothalamic encoding of a persistent defensive state. Nature *586*, 730–734. https://doi.org/10.1038/s41586-020-2728-4.

Kyriazi, P., Headley, D.B., and Pare, D. (2018). Multi-dimensional coding by basolateral amygdala neurons. Neuron *99*, 1315–1328.e5. https://doi.org/10.1016/j.neuron.2018.07.036.

Kyriazi, P., Headley, D.B., and Paré, D. (2020). Different multidimensional representations across the amygdalo-prefrontal network during an approach-avoidance task. Neuron *107*, 717–730.e5. https://doi.org/10.1016/j.neuron.2020.05.039.

Ledoux, J.E. (2000). Emotion circuits in the brain. Annu. Rev. Neurosci. *23*, 155–184.

Levy, D.R., Tamir, T., Kaufman, M., Parabucki, A., Weissbrod, A., Schneidman, E., and Yizhar, O. (2019). Dynamics of social representation in the mouse prefrontal cortex. Nat. Neurosci. *22*, 2013–2022. https://doi.org/10.1038/s41593-019-0531-z.

Liu, X., Ramirez, S., Pang, P.T., Puryear, C.B., Govindarajan, A., Deisseroth, K., and Tonegawa, S. (2012). Optogenetic stimulation of a hippocampal engram activates fear memory recall. Nature *484*, 381–385. https://doi.org/10.1038/nature11028.

Li, Y., Mathis, A., Grewe, B.F., Osterhout, J.A., Ahanonu, B., Schnitzer, M.J., Murthy, V.N., and Dulac, C. (2017). Neuronal representation of social information in the medial amygdala of awake behaving mice. Cell *171*, 1176–1190.e17. https://doi.org/10.1016/j.cell.2017.10.015.

CellPress
OPEN ACCESS

Liu, J., Lyu, C., Li, M., Liu, T., Song, S., and Tsien, J.Z. (2018). Neural coding of appetitive food experiences in the amygdala. Neurobiol. Learn. Mem. *155*, 261–275. https://doi.org/10.1016/j.nlm.2018.08.012.

Mathis, A., Mamidanna, P., Cury, K.M., Abe, T., Murthy, V.N., Mathis, M.W., and Bethge, M. (2018). DeepLabCut: markerless pose estimation of user-defined body parts with deep learning. Nat. Neurosci. *21*, 1281–1289. https://doi.org/10.1038/s41593-018-0209-y.

McGaugh, J.L. (2004). The amygdala modulates the consolidation of memories of emotionally arousing experiences. Annu. Rev. Neurosci. *27*, 1–28. https://doi.org/10.1146/annurev.neuro.27.070203.144157.

O'Keefe, J., and Bouma, H. (1969). Complex sensory properties of certain amygdala units in the freely moving cat. Exp. Neurol. *23*, 384–398. https://doi.org/10.1016/0014-4886(69)90086-7.

Ono, T., Nishijo, H., and Uwano, T. (1995). Amygdala role in conditioned associative learning. Prog. Neurobiol. *46*, 401–422. https://doi.org/10.1016/0301-0082(95)00008-J.

Paton, J.J., Belova, M.A., Morrison, S.E., and Salzman, C.D. (2006). The primate amygdala represents the positive and negative value of visual stimuli during learning. Nature *439*, 865–870. https://doi.org/10.1038/nature04490.

Wang, Y., Fontanini, A., and Katz, D.B. (2006). Temporary basolateral amygdala lesions disrupt acquisition of socially transmitted food preferences in rats. Learn. Mem. *13*, 794–800. https://doi.org/10.1101/lm.397006.

Wolff, S.B.E., Grundemann, J., Tovote, P., Krabbe, S., Jacobson, G.A., Muller, C., Herry, C., Ehrlich, I., Friedrich, R.W., Letzkus, J.J., and Luthi, A. (2014). Amygdala interneuron subtypes control fear learning through disinhibition. Nature *509*, 453–458. https://doi.org/10.1038/nature13258.

STAR★METHODS

KEY RESOURCES TABLE

REAGENT or RESOURCE	SOURCE	IDENTIFIER
Antibodies		
rabbit anti-GFAP	DAKO Logistics Company ApS	N1506; RRID:AB_10013482
Goat anti-rabbit Alexa Fluor 568	Life Technologies Limited	A11011; RRID:AB_143157
Chemicals, peptides, and recombinant proteins		
Neuro-DiO	Biotium	#60015
Experimental models: Organisms/strains		
Lister Hooded Rats	Charles River	603
Software and algorithms		
Spike GLX	https://billkarsh.github.io/SpikeGLX/	N/A
Kilosort 1.0	https://github.com/cortex-lab/KiloSort	N/A
DeepLabCut 2.0.1	https://github.com/DeepLabCut/DeepLabCut	N/A
scikit-learn	https://scikit-learn.org/stable/	N/A
scipy	https://scipy.org/	N/A
Prism 8	GraphPad	https://www.graphpad.com/scientific-software/prism/
Custom analysis code	[Zenodo]: [10.5281/zenodo.6547050]	https://zenodo.org/badge/latestdoi/491890756

RESOURCE AVAILABILITY

Lead contact

Further information and request for resources and should be directed to and will be fulfilled by the lead contact, Cristina Mazuski (c.mazuski@ucl.ac.uk).

Materials availability

This study did not generate new reagents.

Data and code availability

- All data reported in this paper will be shared by the lead contact upon request.
- Original code has been deposited at Zenodo and is publicly available as of the date of publication. DOIs are listed in the Key resources table.
- Any additional information required to reanalyze the data reported in this paper is available from the lead contact upon request.

EXPERIMENTAL MODEL AND SUBJECT DETAILS

Animals

All rats used in these experiments were adult male or female Lister Hooded rats. Rats were housed in large clear plastic cages and maintained on a 12h reverse light/dark cycle (lights on at 21:30) in a temperature and humidity-controlled room. 5 adult male rats received Neuropixels implants. They weighed between 450 and 600g, were 3-6 months of age, and had never been housed with or exposed to female conspecifics prior to the experiment. Prior to surgery, they were housed 2-3 per cage and single-housed after Neuropixels implantation. Implanted rats were fed once per day and maintained at least 90% of their pre-restriction body weight. Both female and male rats were used as social conspecifics. Females were between 4 and 12 months of age to ensure possible sexual receptivity and male conspecifics were between 3 and 6 months. Conspecific rats were housed 2-3 per cage and received ad libitum access to food. All rats (conspecific and implanted) received ad libitum access to water. All animal procedures were conducted in accordance with the UK Animals (Scientific Procedures) Act (1986).

METHOD DETAILS

Neuropixels implantation

To record from BLA neurons, rats underwent stereotactic surgery to implant a Phase 3A Neuropixels probe (Jun et al., 2017) targeting the left BLA. Rats were anaesthetized with isoflurane in O_2 (2-3%) and given pre-op analgesia subcutaneously (Carprieve 5mg/kg) before being placed in a stereotaxic device. All 5 rats were implanted in the left hemisphere (BLA coordinates relative to bregma, −2.8mm anterior, 5mm lateral and -9mm ventral). A stainless steel reference screw located in the right frontal cortex was connected to the ground/reference cable of the Neuropixels probe during surgery. 2-4 additional screws were threaded into the skull to support the anchoring of the implant with superbond and light curable epoxy. The implant was protected with a copper mesh cage that provided physical protection to the probe, and being grounded to the probe/reference screw, shielded against electrical noise. The opening of the copper mesh cage was wrapped with 3M Coban wrap when the probe was not in use.

4 rats received retrievable Neuropixels implants where the Neuropixels probe was dipped into concentrated Di-O (Neuro-DiO, Biotium, 50mM in isopropanol) 1-2 h prior to implantation. Briefly, the Neuropixels probe was epoxied onto a 3-part CNC machined holder that permitted retrieval of the intact probe post-experiment for later re-use. 1 rat received a Neuropixels probe permanently implanted in a custom-designed 3D printed holder. Post-surgery rats received analgesic and antibiotic mixed in strawberry jelly for 3 and 5 days respectively (Metacam 1mg/kg, Baytril 1%). Animals were allowed to recover for 48 h before a test baseline recording and 72 h before full experiments commenced.

Neuropixels probes were retrieved for reuse after the experiment was completed in 4 rats (average duration of recordings was 7-10 days). Briefly, the rat was reanesthetized (isoflurane in O2, 2-3%) and the copper mesh cage surrounding the implant was removed. Since the Neuropixels probe was solely attached to 1 part of the CNC machined holder, we carefully unscrewed this part from the rest of the holder that was affixed to the skull with superbond. The probes were carefully removed, tested, cleaned (1h in 1% Terg-a-zyme in MilliQ, Sigma-Aldrich, followed by 1h in milliQ) and reused (3 probes used in 4 rats). Anaesthetized rats immediately received an overdose of sodium pentobarbital prior to perfusion.

Histology

After completion of the recording session and/or probe retrieval, rats under isoflurane anaesthesia received an overdose of sodium pentobarbital and were transcardially perfused with phosphate-buffered saline (PBS) and 4% paraformaldehyde (PFA). In the 4 rats that received a Di-O dipped Neuropixels probe, brains were transferred to phosphate buffer (50mM PB) and then coronally sectioned in 50um steps while simultaneously imaged using a custom serial two-photon tomography microscope. For the other rat, the brain was transferred to 30% sucrose in PBS for at least 48 h and frozen on dry ice prior to sectioning on a cryostat. Frozen coronal sections cut at 40 um were collected and stained for GFAP expression (primary antibody: DAKO anti-rabbit GFAP 1:500 in PBSGT - 1% normal goat serum and 0.3% Triton-X in PBS; second antibody: goat anti-rabbit Alexa 568 1:500 in PBSGT). Mounted sections were imaged using a Zeiss Axioscan. Regions of the Neuropixels probe within the BLA were determined by manually registering the Di-O or GFAP track onto the Paxinos brain atlas and mapping the coordinate channel locations of the Neuropixels probe onto the track.

Data collection/experimental setup

Neuropixels recordings took place in a custom-made rectangular acrylic box measuring 100 cm × 70 cm with 45cm high walls. The chamber was illuminated by 6 LED photo lights (F + V K320 Lumic Daylight LED Video Light, Wex lighting) for consistent illumination of the space. Continuous video recordings (minimum 30Hz frame rate) were taken with a Basler color video camera (acA2500-60uc Basler Ace USB Camera with Kowa LM8HC camera lens) focused to the floor of the chamber. Neuropixels recordings collected at 30KHz were multiplexed and digitized on the probe and sent via a connected cable to an FPGA board. Extracellular recordings were collected and saved in binary format using SpikeGLX software (https://github.com/billkarsh/SpikeGLX). The video camera was controlled through custom-written software on labview which saved compressed avi video files of the experimental events and sent a 5V synchronizing signal to the Neuropixels board (National Instruments 2018 with NI USB-6000 for 5V pulse generation). To stabilize the Neuropixels cable and headstage and prevent pulling of the delicate Neuropixels flex cable, we 3D printed a custom-designed holder that held and protected the headstage and cable connection. This was secured to the copper mesh cage on the rat's head using alligator clips and counterbalanced with a small weight.

After a baseline recording and habituation to the recording chamber, we started experiments. Rats implanted with Neuropixels probes were connected to the recording apparatus and allowed to freely explore the chamber. The counterbalance was adjusted as needed for the individual rat. During a single experimental day, rats were presented with a battery of different presentations including female conspecifics, male conspecifics, remote-controlled toy mice (HEXBUG 480-4466-00TG12 Remote Control Mouse Cat Toy) and sweetened cooked rice. Following 5 min of baseline recording, each stimulus was placed into the box on the opposite side of the implanted rat and the rat and stimulus were allowed to freely interact. Social interaction was only discouraged when there was danger to the Neuropixels probe (i.e. conspecific rat climbing upon or attempting to chew on the exposed bit of the probe). After 5 min of free interaction with the stimulus, the stimulus was removed from the box during a suitable window (i.e. when there was a break in the social interaction or in the case of food when the rat had finished eating). Recordings were continued for an additional 5 min prior to the chamber being wiped clean of any feces or urine and the start of the next event sequence. In a randomized order, social conspecifics were presented 2-4 times each while non-social toy and food were presented once each. The entire battery of

presentations was then repeated once more in the same order. Rarely, the implanted rat had to be carefully untwisted due to excessive turning in one direction. To do so, between different stimulus presentations, rats were carefully lifted and untwisted to preserve recording integrity. Experiments were repeated for a minimum of 2 days. 1 of the 5 rats did not receive repeat presentations of stimuli and did not have color labelling necessary for multi-animal tracking, therefore the data from that rat was only included in Figure 1.

Single-cell isolation from multi-unit recordings

Neuropixels and video data were saved in a single file each for individual events. Neuropixels data was concatenated across an entire recording day, noisy channels were removed from analysis and the rest of the data was median subtracted to remove artifacts common across the probe. Single units were isolated through Kilosort 1.0 (https://github.com/cortex-lab/KiloSort) and manually curated using Phy or Phy2.0 (https://github.com/cortex-lab/phy). Single units were manually curated according to the following criteria: less than 0.1% of spikes violated the cell refractory period of 2ms, spike waveform was consistent with a single unit, amplitude of at least 40mV and absence of any 50Hz noise in autocorrelogram. Waveform and crosscorrelograms of all nearby units were compared to verify that there were no two clusters corresponding to the same neuron.

Synchronization of video and electrophysiology

During each frame acquisition of video data, the labview program gave a random 80% chance that a 5V pulse would be sent out to the Neuropixels board. The resulting pulse or no pulse was recording by the program along with the relative timing, preserving a copy of the exact synchronizing spike train that represents the camera frames. We extracted the received spike train from the synchronizing channels of the Neuropixels board through a custom-written python program and verified that the pulses received by the Neuropixels board were of the exact number and sequence as the pulses recorded sent by Labview. We extracted the timing of the received pulses by the Neuropixels board, interpolated based on framerate for the 20% of not-sent pulses and used this as the synchronizing index for synchronizing video to electrophysiology.

Tracking from video data

Prior to experiment, the white fur on the tailbase of 4 implanted rats was marked with a purple marker. Conspecific rats also received fur colorings on their right ear, left ear and tailbase with yellow, orange and blue colors, respectively. All conspecific rats received the same color patterns. This color pattern was replicated on the moving toy mouse to approximate the ‘ears’ and tailbase.

All video data was post-processed offline to identify the coordinate positions of all animals and objects in the recording chamber. We trained Deeplabcut 2.0.1 (Mathis et al., 2018) on videos from 3 different recording sessions which contained different implanted rats and different conspecific rats. Using this trained network, we were able to reliably identify 10 different body parts per rat across all videos. On the implanted rat we identified the following: the left and right headstage, the head location, the left, right, and center napes, the left, right, and center mid-back, and the tailbase. On conspecific rats we identified the left and right ears, the nose, the left, right, and center nape, the left, right, and center mid-back, and the tailbase. Deeplabcut tracking of the experimenter-colored body parts had better accuracy, so we thresholded the other body parts per rat to exclude data points where there was an unnatural distance between body parts. We also excluded all points that were under 0.999 probability for the experimenter-colored body parts and 0.9 for the other body parts. Data was then interpolated linearly to fill in gaps.

From the xy coordinates of the 20 tracked body parts, we calculated features relevant to social behavior. The features, calculated independently for the implanted rat and conspecific, are as follows; head velocity, tailbase velocity, spine length, head angular velocity, and tailbase velocity offset by 1s; from both animals: interindividual distance between different body parts (head, tailbase, nape, and back), headdirection from one rat to the other rats different body parts, the difference in head angle between that of the implanted rat and that of the conspecific, the difference in interindividual distance between the two rats’ heads on the one hand, and the implanted rat’s head and conspecific’s tail on the other, the difference in the headdirection between the two rats’ heads and the implanted rat’s head and conspecific’s tail, and the correlation between head and tail positions over 1s. We removed outliers from these features and interpolated any missing data.

Classifying responsivity of individual neurons

We evaluated the response of each individual neuron to each event. Briefly, we calculated the firing rate of each neuron with 1s bins and compared the firing rate from the 5min event period to the firing rate of the immediately preceding 5min baseline period. We computed the receiver operating curve (ROC) and from that calculated the area under the receiver operating curve (auROC) and converted this into a response score ($auROC - 0.5) \times 2$. We assigned a response score of 0 (auROC of 0.5) to neurons that had low firing rates during the baseline and event periods (less than 50 spikes).

To calculate the average response score, we averaged the response score for each of the 4 stimulus types (male, female, food, and object) and set the threshold for responsiveness at +0.2. Neurons that had a response score above 0.2 for more than one modality were classified as multimodal and split into 2 categories – panresponsive (significant scores on both social and non-social stimuli) or non-social/social. A large proportion of units exceeded the threshold on only one specific stimulus type (event-specific) and these were classified based on their stimulus preference. Units that did not increase firing in response to any stimulus presentation were further split into 2 groups (non-responsive and firing decreases). Neurons that decreased firing had average response scores less than -0.2 for at least one modality.

CellPress
OPEN ACCESS

Classification of putative pyramidal neurons and putative interneurons

We calculated the waveform for each recorded neuron by extracting the raw voltage trace from 500 randomly selected spiketimes per neuron. We averaged these voltages together and calculated the time delay between the trough and peak. A small fraction of waveforms had inverted waveforms (where the peak voltage precedes the trough) and these were excluded from the analysis and classified as irregular waveforms. To divide the rest of the neurons into putative pyramidal or interneurons we plotted the peak to trough distance against the baseline firing frequency from the first 5 min of each recording day. We divided the results into 2 groups using K-Means clustering, with putative pyramidal neurons having longer peak to trough to distances and lower baseline firing rates and putative interneurons having shorter trough to peak distances and higher firing rates.

Population correlation of response scores

To calculate the correlation between population response scores across different stimulus presentation, we calculated the population vector for all responsive neurons (multimodal and event-specific) for each stimulus presentation. To combine population vectors across different rats, we defined social presentations (e.g. female1 and female2) as being the first 2 conspecifics of the same sex presented. If there was also a third female or male conspecific presented to that rat, they were not shown in the heatmap, but included in the quantification. We calculated the Pearson's correlation between the calculated population vectors.

Decoding stimulus identity from neural activity

To test whether the population response score accurately represents the stimulus identity, we used Linear Discriminant Analysis (LDA, python package Sci-kit learn). For each rat, we trained the LDA decoder on all the population response scores of all the stimulus presentations except one and then tested on the omitted dataset (leave-one-out method). We repeated this until we had a predicted stimulus type of each stimulus presentation and calculated the accuracy of these labels against the correct labels, giving one score per rat. We then repeated 500x with shuffled labels and calculated the control score per rat.

To calculate how the number of neurons used in the decoder affected performance, we trained the LDA decoder on a random selection of neurons at increasing numbers (from 2 neurons to the maximum number of neurons per rat, 500x per neuron number selection). We then repeated this analysis, but instead of using all neurons in the dataset we omitted specific classes of neurons (pan-responsive, event-specific and nonresponsive). We calculated the change in performance of the decoder for each rat by subtracting the performance using all neurons from the performance with the omitted neurons controlling for neuron number.

To calculate how the location of neurons affect the accuracy of the LDA decoder for social events, we recalculated the accuracy using the leave-one-out method for all social events but only included neurons from the same rat located within 500um of each other, with a minimum of at least 10 neurons included in the dataset.

To calculate how firing around the time of stimulus contact affected the performance of the LDA decoder, we used the leave-one-out method described above to calculate the accuracy of an LDA decoder trained on a shifting window of 10s of neuronal firing data from 30s before stimulus contact to 30s after contact. We compared the accuracy per rat in 5s epochs from 10s before stimulus contact to 5s after.

Quantification of differences in firing onset and firing offset

We calculated firing rate data at event start with bins of 100ms per neuron. We z-scored the firing rate to the baseline firing rate per event. For visualization across different events, we time-warped individual z-scored traces using Spline Interpolation into a common timeframe. All quantification was performed on the non-interpolated raw z-scored data.

To calculate whether individual units within a specific trial started firing at similar times, we calculated the time delay to peak firing per unit after stimulus presentation but before direct contact. We calculated the Euclidian distance between these values for all units within a single trial. We compared this to the Euclidian distance of the time delays between the firing onsets of the same unit within different trials.

To calculate aftereffects, we once again took z-scored firing rate data from individual stimulus presentations and sorted based on largest average z-score difference in the post-stimulus presentation period relative to baseline. We calculated the proportion of neurons with an average z-score above 1 in 10s bins for 3 min after stimulus removal.

To test whether neural activity before and after interaction with a stimulus can decode stimulus identity, we trained an LDA classifier on response scores during the event period. We calculated the response score from 2 min before event start to 2 min after event stop in 10s bins and testing decoding performance across this binned data.

Automatic classification of behavior from video data

To facilitate the analysis of whether neurons respond to specific social behaviors, we trained a support-vector classifier on 7 videos of manually annotated social behavior. We annotated videos frame-by-frame with one of 7 different labels (non-social, head-head, head-tail, approach, following, conspecific-initiated contact and other social) as detailed.

Non-social: No social engagement from either the implanted animal or conspecific.

Head-to-head: Implanted animal and conspecific facing each other and making direct contact with snout or whiskers

Head-to-tail: Implanted rat less than one head's distance from the ano-genital region of the conspecific

Approach: Implanted rat moving towards a stationary conspecific

Following: Implanted rat greater than one head's distance from the conspecific with both animals facing and moving in the same direction

Conspecific-initiated contact: Implanted rat is turned away from the conspecific, while the conspecific is engaged with and touching the implanted rat (usually sniffing the implanted rat's backside or flank).

Other Social: All other social engagement, which includes but is not limited to social grooming, boxing, flank nipping, mounting.

Using the trained SVC classifier, we were able to automatically and reliably identify social and non-social periods as well as specific social behaviors including head-to-head contact, head-to-tail contact, approach, following and conspecific-initiated contact. We used 30 features calculated from the xy coordinates for the 20 body parts on the 2 different rats and trained the support vector classifier with rbf kernel. Using 5-fold cross-validation, initial tests revealed that the SVC classifier correctly labeled data with 97% accuracy. We retested the SVC classifier by omitting one out of 7 of the manually annotated datasets in each iteration and determined that the SVC classifier had an accuracy of approximately 90% for frame-by-frame annotation prediction. We then trained the rest of the collected videos on the trained SVC classifier of the manually annotated data and post-processed the bouts by removing behavior gaps shorter than 0.25ms and behaviors that lasted for less than 5 frames.

We performed a simpler automatic classification of non-social videos. For interaction with a moving toy object, we calculated the optimal pixel threshold for differentiating social contact vs no-contact from the automatically identified social video data and applied this threshold to the videos depicting toy interaction. For the food interactions, given that the rat was stationary while eating the sweet rice, we used the video and tracking data to draw an ROI at the position where the rat was eating the sweet rice and used this to calculate moments of eating vs not eating the sweet rice.

Calculating unit response to behavior

To calculate whether individual units increased or decreased their firing in response to specific behaviors, we compared firing rates of each neuron for periods of interaction to periods of non-interaction that occurred after the point of first contact with the stimulus. We calculated the auROC and response score for the comparison of these two distributions for each individual event and calculated the average response by averaging the response score for all the events that the unit responded to (e.g. interaction score for event-specific units was calculated only from those events, etc.). Units that had an interaction score greater than +0.2 or less than −0.2 were classified as increasing or decreasing firing in response to interaction.

To calculate whether units experienced time-locked increases or decreases to specific behaviors, we extracted the timestamps of all individual behaviors (head-head, head-tail, approach, following, conspecific-contact) that lasted more than 1s. We calculated the peri-event stimulus histogram for each behavior by extracting all spike times from five seconds before to five seconds after and summed the spikes binned in 100ms. We z-scored the resulting average traces per neuron to the first 3 s and considering any neuron showing a z score above +5 or below −5 to be responsive to that particular behavior.

Decoding behavior from neural activity

We took the z-scored population data with labels for interaction or non-interaction per individual event and calculated the ability of an LDA decoder to predict whether the animal was interacting or not interacting with the stimulus using 5-fold cross validation. Each dot represents a different event comparing the predicted score against the actual score using ROC analysis. We reran the analysis with different numbers of neurons as well as separating the events based on stimulus type.

Cross-correlation analyses

To identify pairs of cross-correlated neurons across the entire recording session, we calculated the cross-correlogram for every simultaneously recorded neuronal pair with a resolution of 0.25 ms. To determine significance, we identified cross-correlograms with increased firing that exceeded 3 standard deviations. We used a peak detection algorithm on the cross-correlogram between −5 and +5 ms (scipy.signal.find_peaks, height = 3 standard deviations above mean, width = 0.75 ms) and filtered the dataset to only include cross-correlograms where the peak occurred after 0 ms and the peak width did not start before 0ms (representing directional putative monosynaptic connections). We excluded neuronal pairs where either the projection or target neuron fired less than 100 spikes or when the peak firing in the cross-correlogram was lower than 20 spikes. To determine neuronal connectivity during the first presentation of each stimulus type, we determined how many of the previously identified cross-correlated neurons showed significant cross-correlations during the baseline, event or post-event period for the 4 stimuli using the same criteria as above.

To determine whether connectivity between certain types of neurons was overrepresented in the dataset compared to chance, we calculated the actual incidence of different connection types in the dataset and compared that to theoretical incidence of the connection type if the neurons were randomly connected (1000x random sampling between the observed projection and target neurons).

To calculate connectivity strength, we determined the number of spikes that occurred during the detected cross-correlogram peak and divided that by the total number of spikes fired by the target neuron during the epoch. To determine whether changes in connectivity occurred within individual neuron pairs, we identified neuronal pairs that showed connectivity between two consecutive epochs (baseline and event or event and post-event) and compared their connectivity strengths.

Tracking neurons across two days of recording

To track neurons across two days of recording, for each neuron we calculated the normalized mean waveform across 20 consecutive Neuropixels channels. For each neuron on day1, we compared this waveform to the normalized, mean waveform from all neurons within +/− 5 channels on day2. Briefly, for all possible day2 neurons, we calculated the structural similarity index (SSIM – a measure of image similarity, scikit-image) from the centered waveform heatmaps and the per channel pearson correlation of the waveform trace. We visually verified that the neuron pair with the highest SSIM and pearson correlation obviously correlated between day1 and day2. This resulted in 121 neurons tracked between day1 and day2.

Speed correlation

To determine whether the firing rate of individual neurons was correlated with animal speed, we binned neuronal firing data in 50ms bins and cross-correlated with the speed vector during baseline, event and post-event periods. We calculated the average cross-correlated profile during these 3 time periods and classified neurons as positively or negatively correlated with speed based on whether the average cross-correlation at time 0 was higher or lower than the cross-correlation between −20s and −10s. As previously done with decoding behavior from neural activity, we took the z-scored population data alongside labels for rest or movement per individual event and calculated the ability of an LDA decoder to predict whether the animal was moving or not moving using 5-fold cross validation.

QUANTIFICATION AND STATISTICAL ANALYSIS

Statistics

The data in bar graphs are plotted as mean ± SEM where SEM refers to standard error of the mean. Data on box-plots correspond to the median and 25th-75th percentiles. Statistical analysis was performed in PRISM (Graphpad) software or using stats-models Python packages and individual statistical tests are as noted in the figure legends. Differences between two groups were assessed using non-paired Student's t-test or Kruskal-Wallis test with Dunn's correction where normality could not be assumed. Differences within a group were assessed using paired Student's t-test or Kologrov-Smirnov test where normality could not be assumed. Comparisons between 3 or more different groups were assessed using 1-way analysis of variance (ANOVA) with Tukey's test and 3 or more comparisons within-group were assessed using repeated-measures 1-way ANOVA with Geisser-Greenhouse correction and Tukey's test. Repeated-measures two-way ANOVA with Geisser-Greenhouse correction followed by Sidak's test was used to compare how different groups evolved over time (i.e. event-specific or panresponsive neurons across time). To evaluate the association between two variables, Pearson correlation coefficient was used. $p < 0.05$ was considered statistically significant and p values are denoted by the number of stars, *, ** representing $p < 0.05$, $p < 0.01$, respectively.

Cell Reports, Volume *39*

Supplemental information

Representation of ethological events by basolateral amygdala neurons

Cristina Mazuski and John O'Keefe

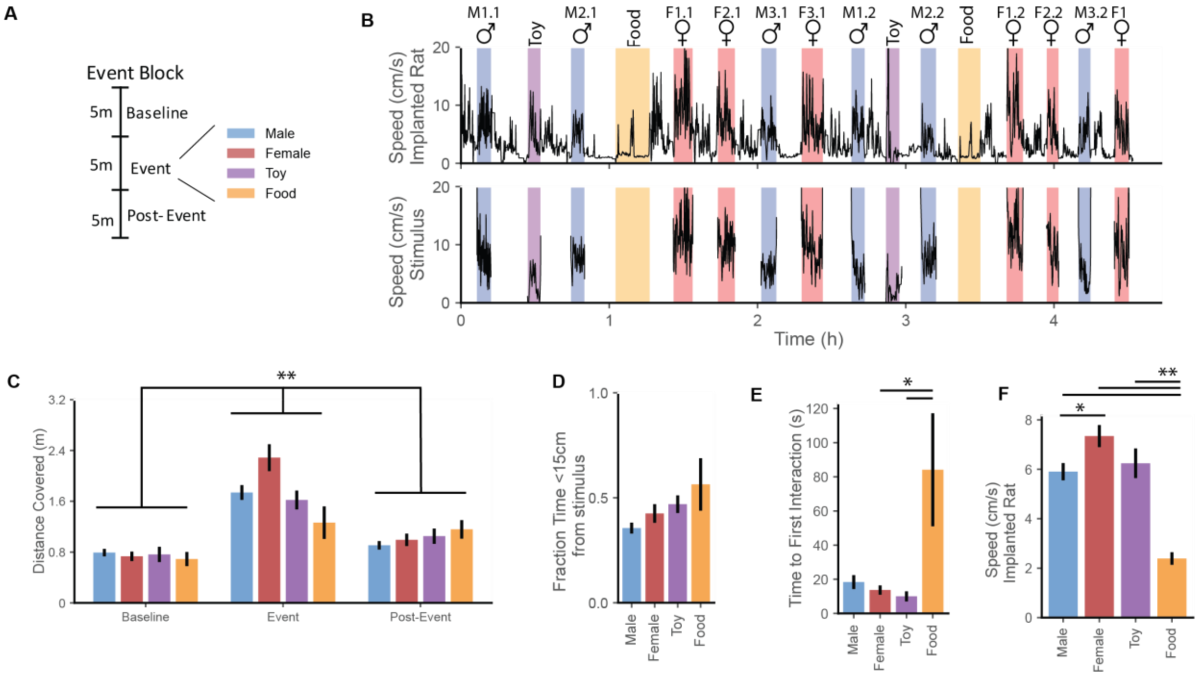

Fig S1. Presentation of a battery of social and non-social stimuli and resulting behavior. Related to Figure1.

(A) Representation of a single event block and different stimuli or event-types presented. During a recording session (representative example in B), an implanted rat was presented with up to 16 different event blocks. The implanted rat was continuously tracked throughout the entire recording session and non-stationary stimuli (male conspecifics, female conspecifics, moving toy) were tracked when present. C) The total distance covered by the implanted rat increases during interaction with stimuli, but there are no consistent differences between different types of stimuli. (**$p<0.001$ for time, $p=0.08$ for event-type, repeated measures 2-way ANOVA) D) Implanted rats spend similar amounts of time within 15cm of stimuli indicating that there was not an overall preference for one type of stimuli over another. ($p = 0.16$; 0.36 ± 0.03, 0.42 ± 0.04, 0.47 ± 0.04, 0.56 ± 0.12; Kruskal-Wallis test) E) Rats take longer to approach and interact with food than moving stimuli (*$p<0.05$; 18.39 ± 4.05, 13.7 ± 2.7, 10.01 ± 2.95, 84.10 ± 33.1; Kruskal-Wallis test). F) There are large differences in speed when the rat is interacting with stationary versus non-stationary stimuli (food vs. males, females or moving toy, **$p<0.01$,). Within the different non-stationary stimuli, the implanted rat moves at slightly higher speeds when interacting with female vs. male conspecifics (*$p<0.05$), but speed during interaction with a moving toy is not different to speed during social interaction (toy vs. male, $p=0.96$; toy vs. female $p=0.42$; 5.90 ± 0.35, 7.34 ± 0.45, 6.24 ± 0.60, 2.40 ± 0.25; Ordinary 1-way ANOVA). Values for plots D-F from male (N=27), female (N=17), toy (N=8), and food (N=8) events respectively.

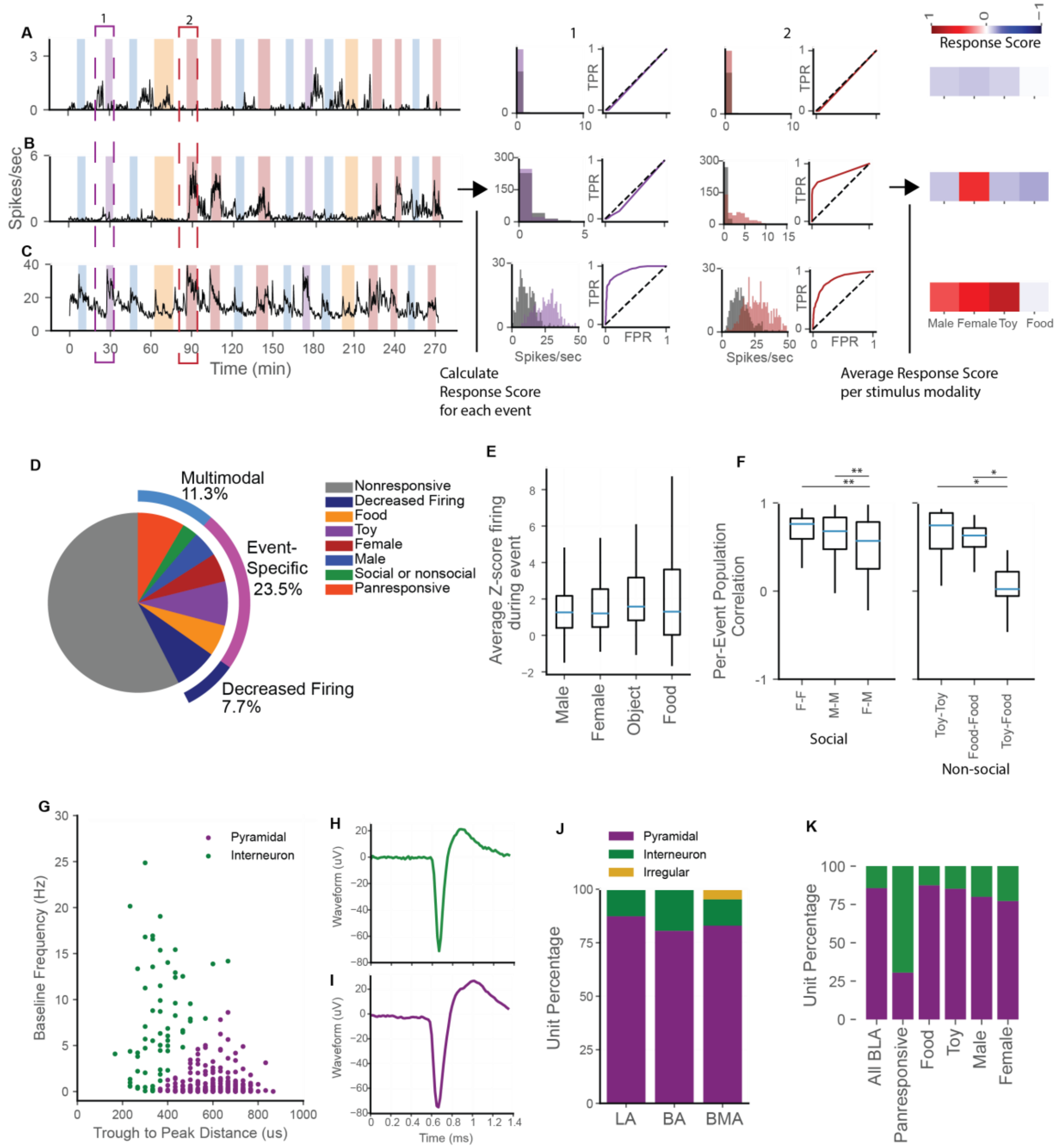

Fig. S2. Calculation of response scores for presentations of different stimuli and neuronal properties. Related to Figure1.

For each neuron, we first computed the receiver operating characteristic curve (ROC curve) and calculated the area under the curve (auROC, between 0-1). We averaged together the auROCs for each event (male, female, toy, food) and converted this into a response score (between -1 and 1). Neurons were classified based on their response scores on each of the four events. A) A representative unresponsive neuron that does not have a positive response to any event. B) A representative event-specific neuron, in this case firing only to females. These neurons show a positive response score to only one event. C) A representative panresponsive neuron, which

shows increases in firing to both social (male/female) and non-social (toy/food) stimuli. Dashed boxes show the periods over which the ROC was calculated for the toy and the first female events. D) The percentage of all BLA neurons with consistent responses to different events. E) There were no differences in the average z-scored firing activity of responsive neurons across different event types. ($p = 0.40$; Ordinary one-way ANOVA; N = 306, 231, 134, 109 trials for male, female, toy and food respectively). F) Population correlation between social (** $p< 0.01$; N = correlation of paired social events, 30, 79, 112 for F-F, M-M and F-M, respectively; Kruskal-Wallis) and non-social (*$p<0.05$; N = correlation of paired non-social events, 4, 4, 16 for toy-toy, food-food, toy-food respectively, Kruksal-Wallis). G) Neurons were classified using K-Means clustering as putative pyramidal or putative interneurons based on their baseline firing frequency (first 5 min of the recording) and the distance between the trough and peak waveform. A representative putative interneuron is shown in (H) and a representative pyramidal neuron shown in (I). J) Small regional differences existed between different BLA nuclei with fewer interneurons being present in the LA than the BA/BMA. Irregular waveforms (where the only waveform peak precedes the waveform trough) were only present in the BMA and were excluded from the analysis in (G). K) Panresponsive neurons are more likely to be putative interneurons than other event types.

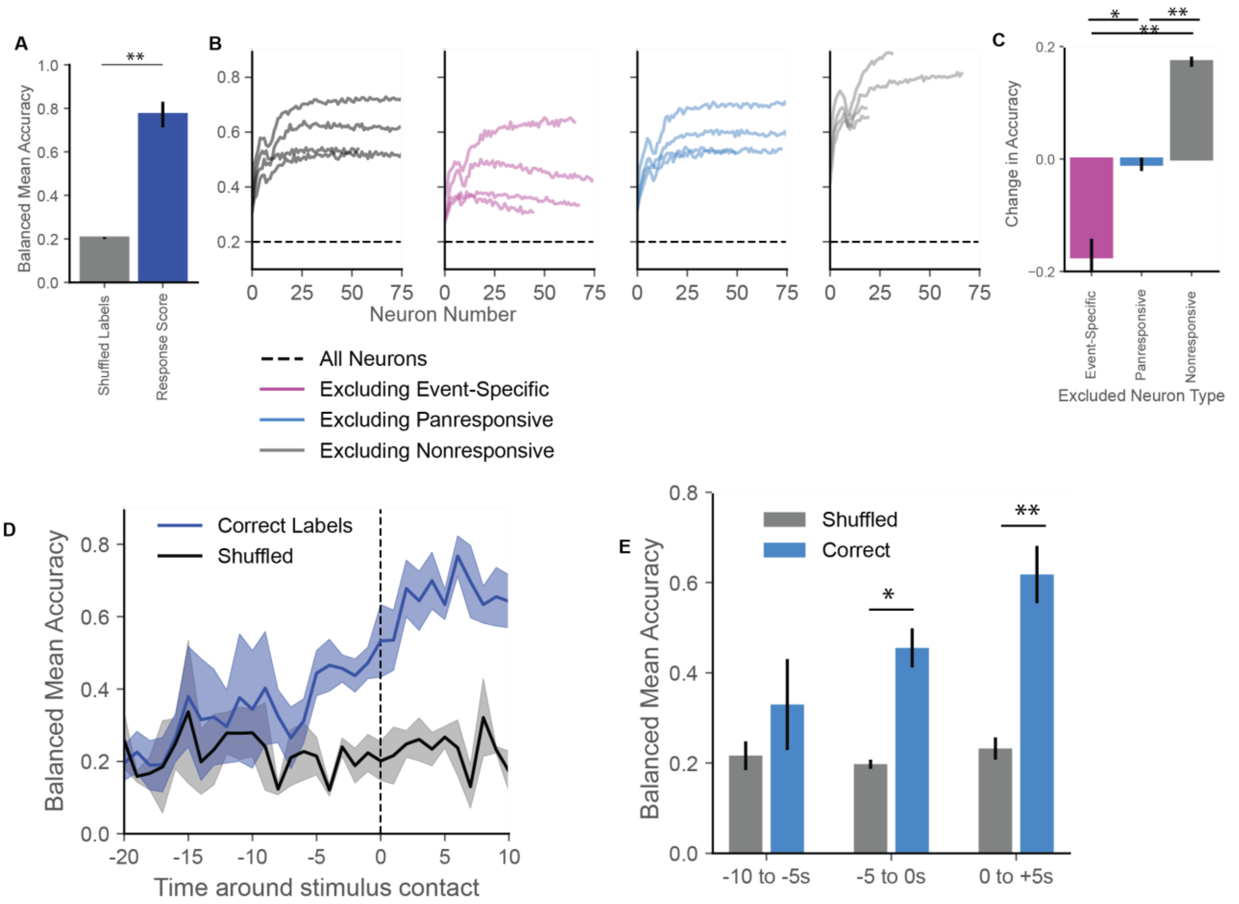

Fig. S3. Reliable decoding of stimulus identity. Related to Figure1.
A) Stimulus identity can be reliably decoded from neuronal response scores using LDA analysis. (** $p<0.01$; 0.20 ± 0.004, 0.78 ± 0.08 for shuffled versus actual, mean ± SEM; N = 4 rats, Student's paired t-test) B) Relatively few neurons are needed for reliable stimulus identity decoding. By excluding specific neuron types (event-specific, panresponsive or nonresponsive) we can measure the relative contribution of each neuron class (each line represents a separate rat, see methods). C) Event-specific neurons carry more information about stimulus identity than panresponsive neurons, which in turn carry more information than nonresponsive neurons. Removing event-specific neurons impairs performance, while removing nonresponsive neurons improves performance (* $p < 0.05$, **$p < 0.01$; -0.17 ± 0.03, -0.01 ± 0.01, 0.17 ± 0.01 for excluding event-specific, panresponsive or nonresponsive neurons respectively, mean ± SEM; N = 4 rats; repeated measures one-way ANOVA). D-E) To determine whether contact with a given stimulus affected decoding of stimulus identity, we reran LDA using mean firing frequency over 10s around the start of direct contact with the stimulus. Trial identify could be reliably decoded from neuronal activity starting from approximately 5s before direct contact with the stimulus. (-10 to -5s: p=0.52; -5 to 0s: *$p<0.05$; 0 to +5s: **$p< 0.01$; repeated measures 2-way ANOVA, N = 4 rats)

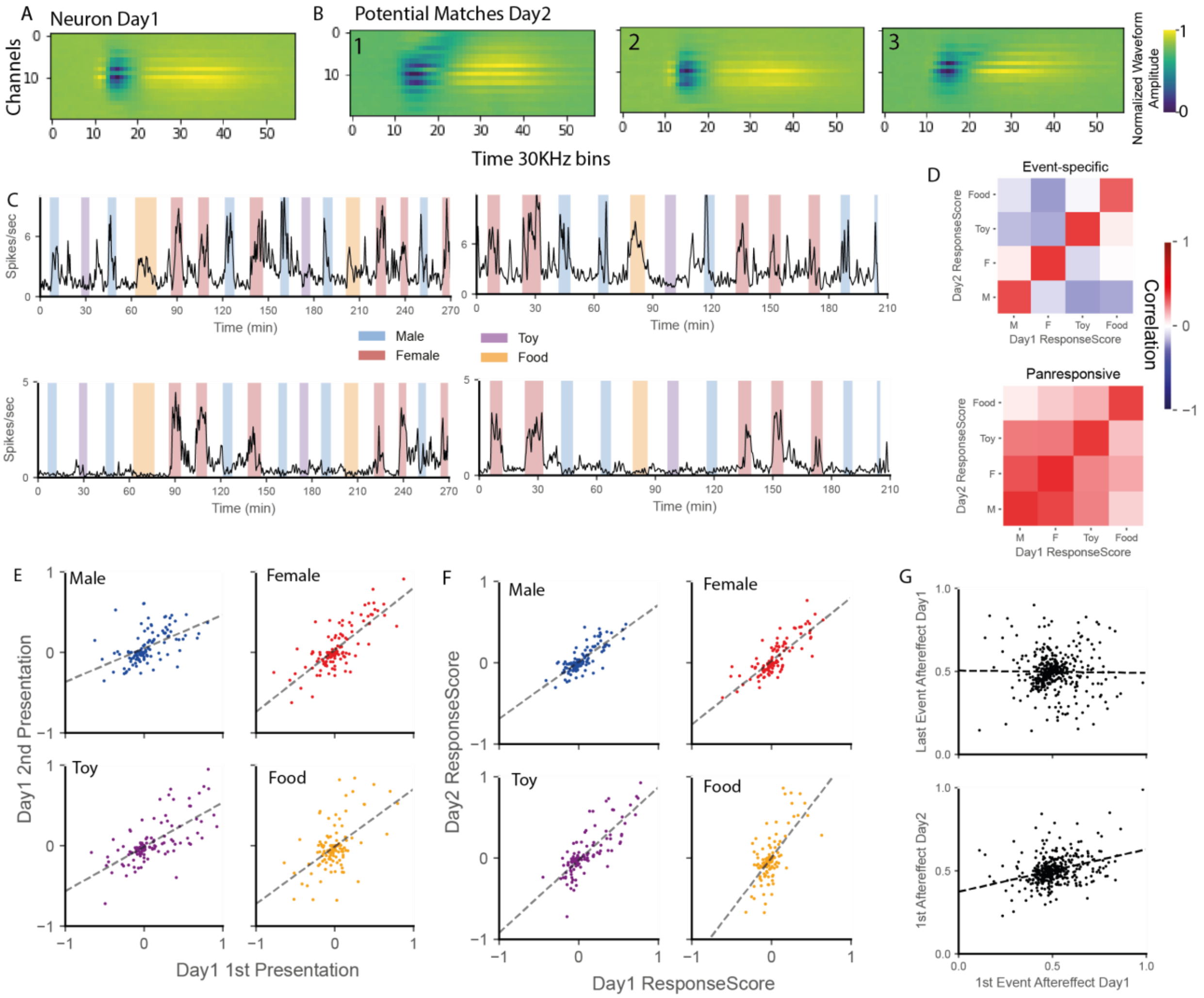

Fig. S4. Stable representation of ethological stimuli across two days. Related to Figure1. 121 out of 426 neurons were tracked across two days of recording. A) We compared the normalized waveform across multiple Neuropixels channels from day1 of the recording to potential matches on day2 (B, see methods). In the example in A-B, the neuron on day1 is ultimately paired with neuron2 on day2. C) Representative examples of a panresponsive (above) and event-specific neuron (below) showing stable coding of different event-types across two days. D) The population vector correlation of the response scores on day1 and day2 for event-specific and panresponsive neurons show stable representation. E) Individual neuronal response scores for single event presentations are highly correlated within a single day of recording (**$p<0.01$; $r^2 = 0.52, 0.77, 0.68, 0.54$ for male, female, toy and food respectively; N = 121 neurons) F) Neuronal response scores per event-type are highly correlated between 2 days of recording (**$p<0.01$; $r^2 = 0.79, 0.82, 0.79, 0.66$ for male, female, toy and food respectively; N = 121 neurons). G) Aftereffect strength is not correlated between first and last event presentations within a single day (top panel; within-day aftereffect correlation: $p=0.78$, $r^2=-0.01$), but shows correlation across 2 days of recording (bottom panel; across two days aftereffect correlation: **$p<0.01$, $r^2 = 0.34$). N=426 aftereffect auROC values for all event-types.

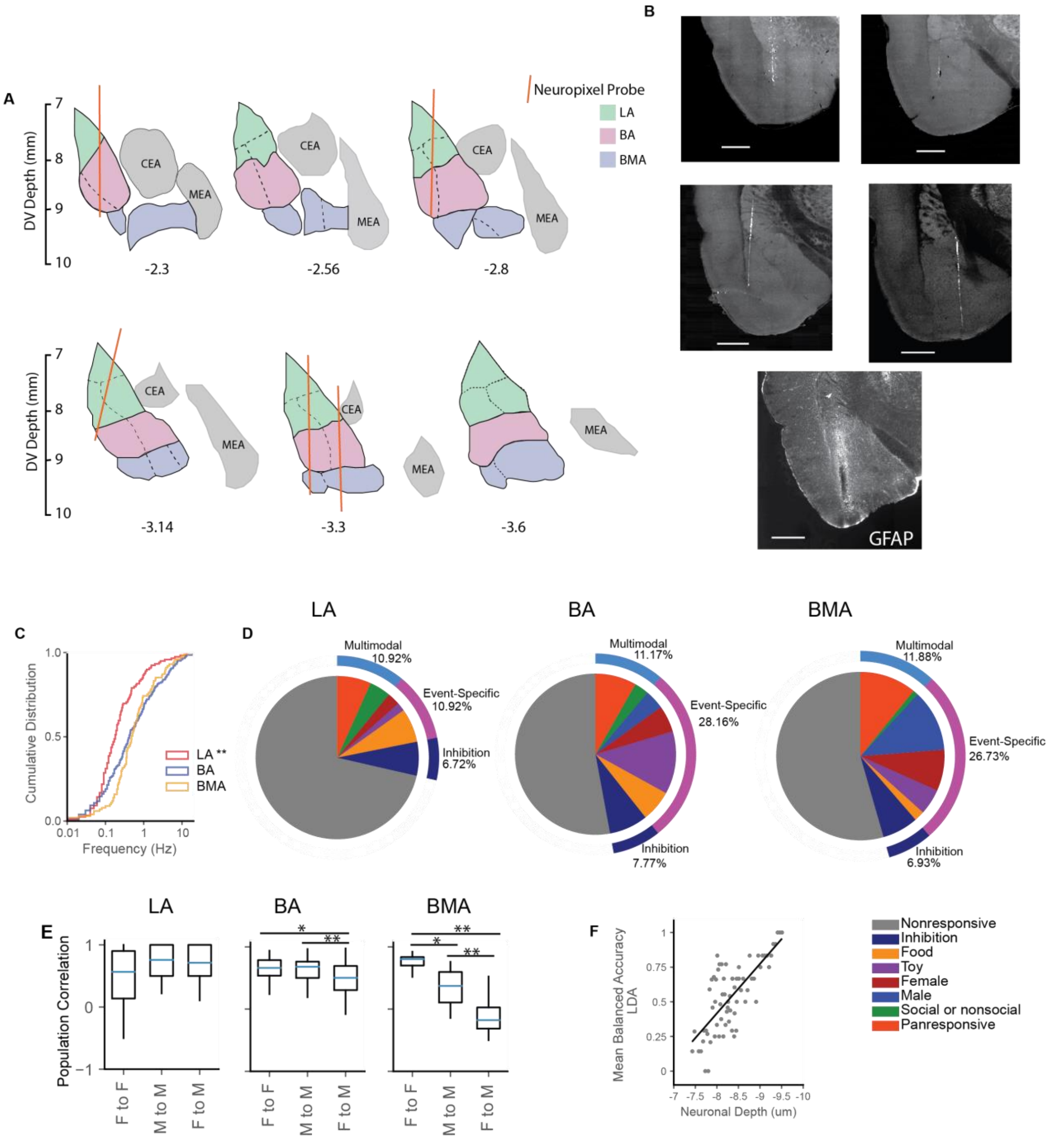

Fig. S5. Histological Reconstruction of Neuropixel probe implants. Related to Figure1. A) Neuropixel tracts were manually registered to a Paxinos rat brain atlas to determine which probe channels were located in specific BLA nuclei. B) Histological images from 5 rats used in this study showing the Neuropixel probe entering the BLA either using the Di-O tract or GFAP staining. (N=119, 206, 101 neurons for LA, BA, and BMA respectively, scale bar = 1mm) C) LA has lower baseline firing rates than the BA and BMA. (**p<0.01 LA vs. BA and BMA; 0.76 ± 0.18, 2.0 ± 0.31, 1.4 ± 0.24, baseline firing rate from LA, BA and BMA neurons, respectively, mean ± SEM; Kruskal-Wallis test). This is consistent with the presence of fewer interneurons in that region (S2). D) LA (left) has fewer responsive neurons than BA (middle) and BMA (right)

and neuronal selectivity to specific event-types varies across region with more non-social responses (i.e. event-specific food or toy) in the BA and more social responses in the BMA (i.e. event-specific male or female). E) Discrimination between male and female conspecifics is present in the BA and BMA but not in the LA (LA, $p = 0.62$; ordinary one-way ANOVA; BA, *$p < 0.05$, **$p < 0.01$; ordinary one-way ANOVA, BMA *$p < 0.05$, **$p < 0.01$; Kruskal-Wallis test, N(LA) =36, 12, 32, N(BA) = 24, 58, 84, N(BMA) = 15, 15, 36 for event-pairs F-F, M-M and F-M, respectively). F) Decoding of social stimulus identity changes along the dorsal-ventral axis, with better discrimination of social conspecifics at deeper co-ordinates. (accuracy of decoding social events, **$p < 0.01$, $r^2=0.56$, pearson correlation).

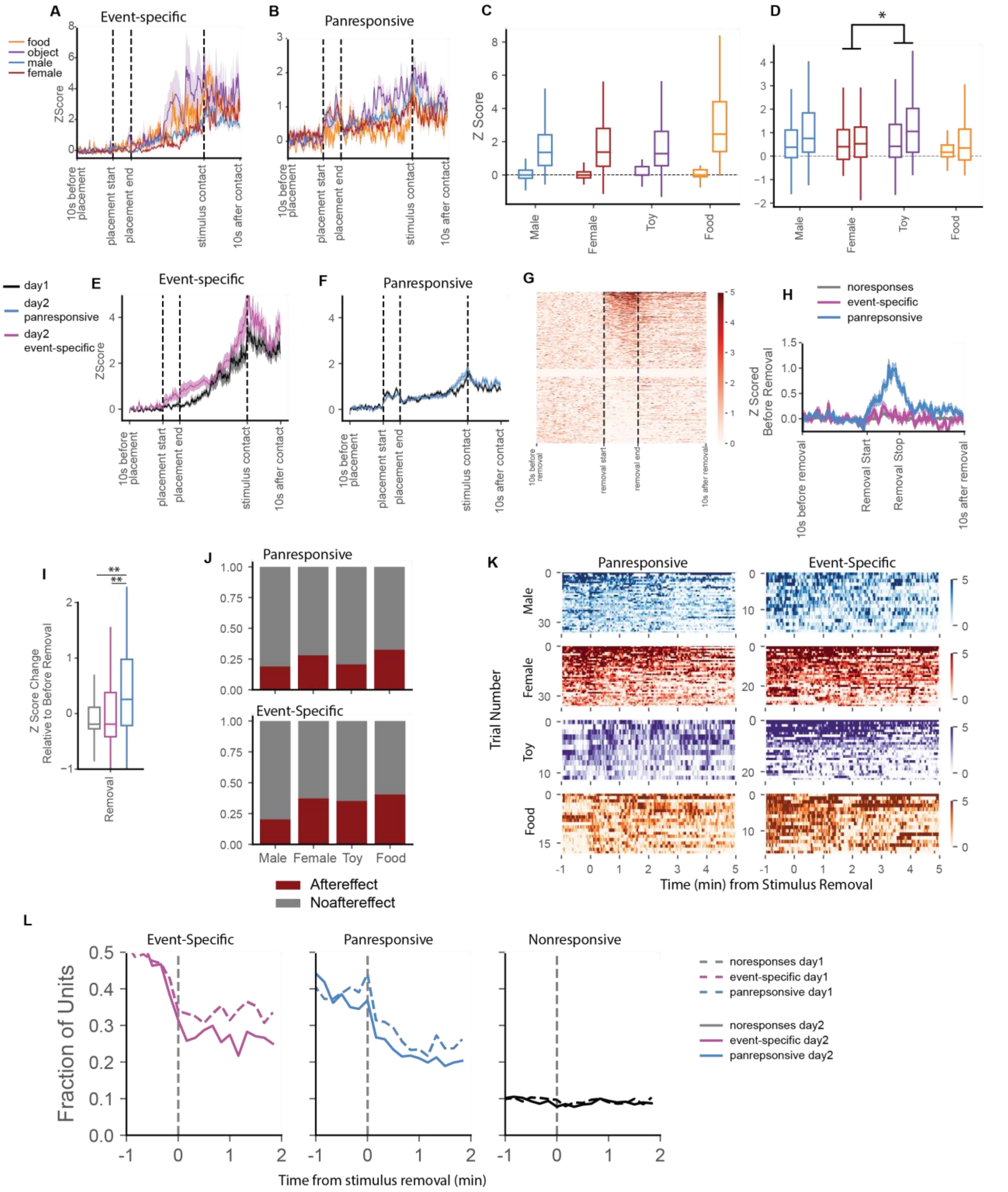

Fig. S6. Similarities in firing onset between different events and across different recording days. Related to Figure2.

A) Event-specific and B) panresponsive neurons show similar time-courses irrespective of the stimulus identity, suggesting that the increases in firing are not due to the presence of a specific sensory cue but to occurrence of the event. The total level of activation during the stimulus presentation and stimulus contact (left and right boxplot for each modality, respectively) was

similar across all stimulus modalities in C) event-specific ($p=0.08$ for event-type differences, **$p<0.01$ for differences across time, N = 222, 174, 152, 92 for event-specific male, female, toy and food responses, respectively, repeated measures 2-way ANOVA) and D) panresponsive neurons, with the exception of slightly higher firing within panresponsive neurons in response to toy stimuli compared to female conspecifics (*$p<0.05$ for event-type differences, **$p<0.01$ for time differences, N = 390, 232, 116, 108 for panresponsive trials during male, female, toy and food events, respectively, repeated measures 2-way ANOVA) E and F) There were no differences in the timecourse in event-specific or panresponsive neurons across the two recording days. G) Z-scored firing data from 10s before stimulus removal to 10s after removal. Data were timewarped between removal start and removal stop to enable comparison between different trials. H-I) Only panresponsive neurons show a transient increase in firing during the removal of the stimulus (**$p<0.01$, N = 1239, 280, 389 for nonresponsive, event-specific and panresponsive trials, respectively; Kruskal-Wallis). J) The proportion of panresponsive and modality-specific neurons that show aftereffects is similar across different stimulus types. K) Z-scored images show after-effect producing neurons that continued to fire for up to 5 min following stimulus removal. L) The proportion of aftereffects found in event-specific and panresponsive neurons decreases slightly on the second day of recording.

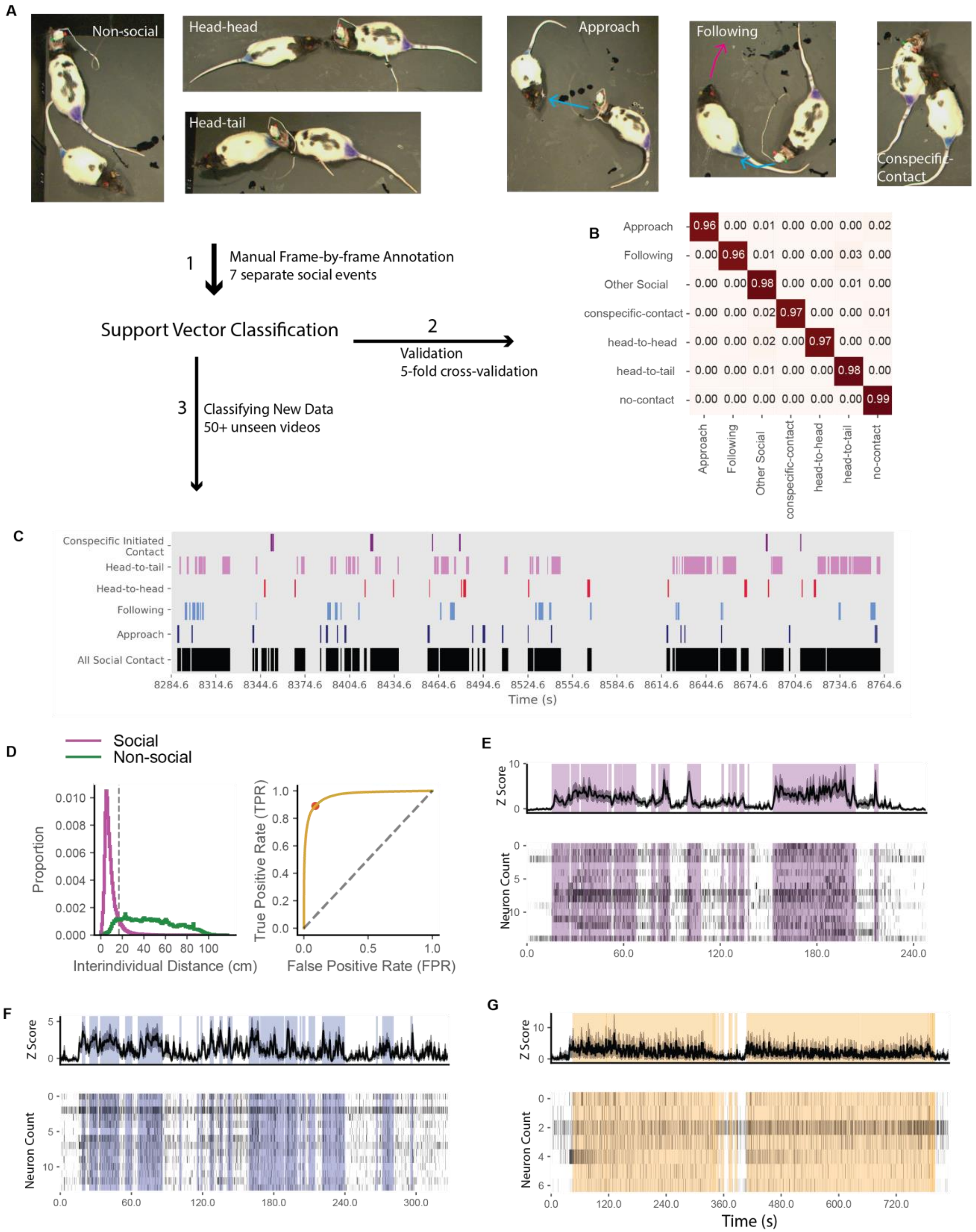

Fig. S7. Automatic Classification of Social Behavior. Related to Figure3.

A support vector classification was used to standardize the analysis of social behavior across all social interactions. A) 7 social interaction events (4 male-female events and 3 male-male

events) were manually annotated frame-by-frame for one of 7 distinct behaviors (see methods for description of the behaviors). Pictured are non-social behavior and 5 distinct social behaviors. B) We trained the SVC classifier on the manually annotated behavior using the features extracted from the automated tracking. The SVC classifier was validated using 5-fold cross validation, which yielded balanced accuracies of 0.97. (see methods for a full-description of the analysis). C) We used the trained SVC classifier to automatically classify social behavior in previously unseen videos. Using this classification method, we identified two layers of behavioral activity from each social event. #1 – whether the animals were interacting (social) or not interacting (non-social) and #2 – the timing of 5 distinct social behaviors (sensory behavior – i.e. head-head or head-tail, movement behavior i.e. approach or following and passive i.e. conspecific contact). D) To automatically calculate stimulus-interaction in nonsocial events, we calculated the optimal interindividual distance threshold from all social trials using ROC analysis (see methods, threshold = 16.8cm). This threshold was also applied to non-social trials to identify behavioral interactions with the stimulus. E-G) Interaction-modulated neurons across different stimulus presentations illustrate the presence of single-cell and neuronal populations that reliably track engagement in both social and non-social stimuli E, toy; F, males; G, food.

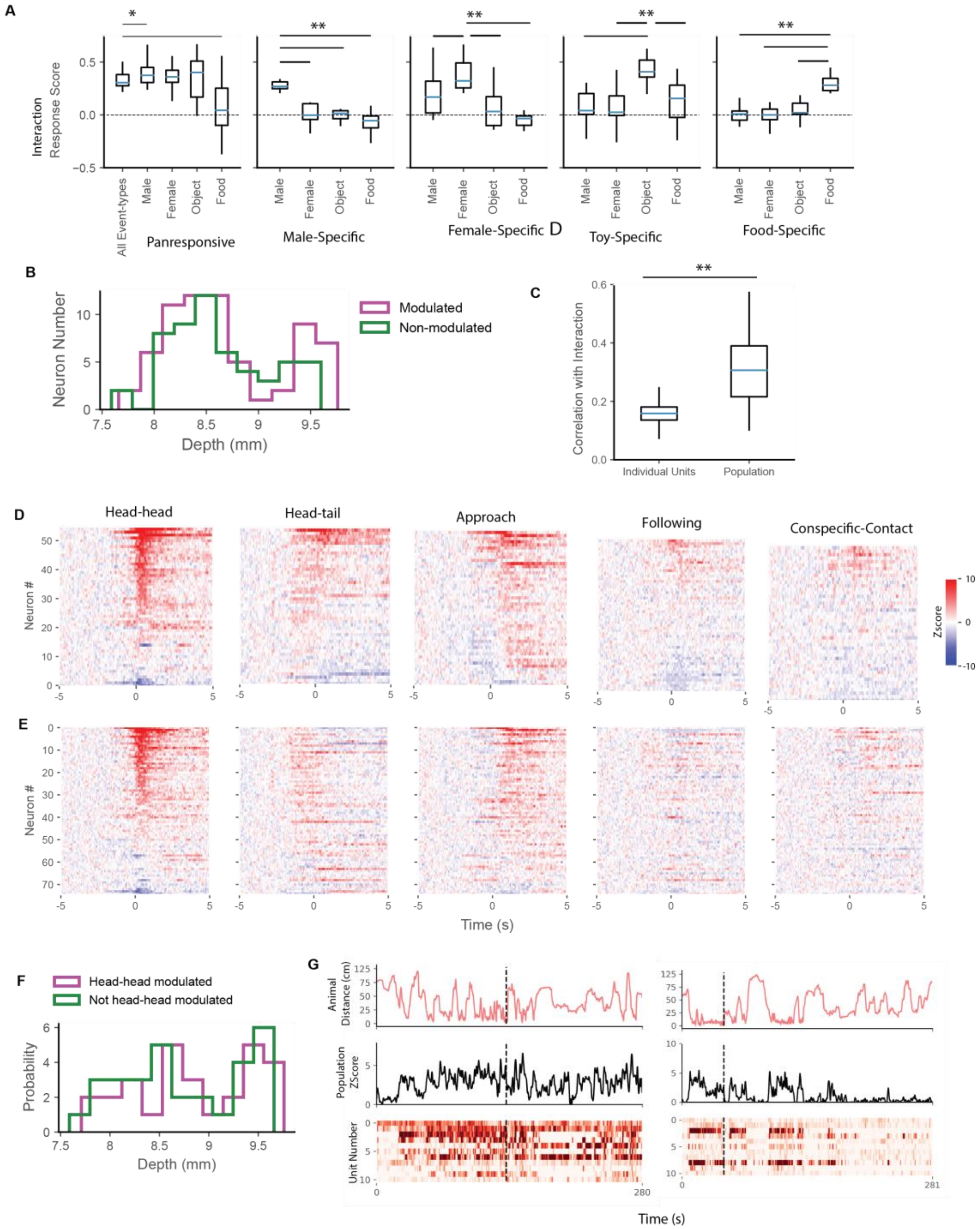

Fig. S8. Properties of neurons modulated by interaction with stimulus. Related to Figure3.

A) Interaction-modulated event-specific neurons only show modulation by the preferred type of stimuli. For example, male-specific neurons do not show the same levels of modulation to female, toy or food stimuli. (**$p < 0.01$, N = 8, 13, 15, 11 for male, female, toy and food exclusive engagement-modulated neurons, repeated-measures 1-way ANOVA). Panresponsive neurons show mixed selectivity (*$p < 0.05$, N = 15). B) Within responsive neurons, interaction-modulated neurons are not anatomically clustered compared to non-interaction modulated neurons. C) The population vector of interaction-modulated neurons is more highly correlated

with behavior compared to the average individual units per event (**p < 0.01, N = 58 events, unpaired Student's t-test). D) A proportion of neurons show responses to specific social behaviors (each heatmap is sorted according to maximal response). More units respond to head-head, head-tail contact and approach than to following and conspecific-contact. E) Some units show selectivity to multiple social behaviors (all heatmaps sorted by the individual neuron's response to head-head contact). F) Head-head modulated neurons are not anatomically localized any more than non head-head modulated neurons. G) Responsive units during interaction with a female conspecific do not show strong modulation as a result of mating (black dashed line) in these two example encounters. While activity appears to transiently dip after mating, this can be explained by the end of active interaction with the female conspecific as illustrated by the increase in animal distance immediately after mating.

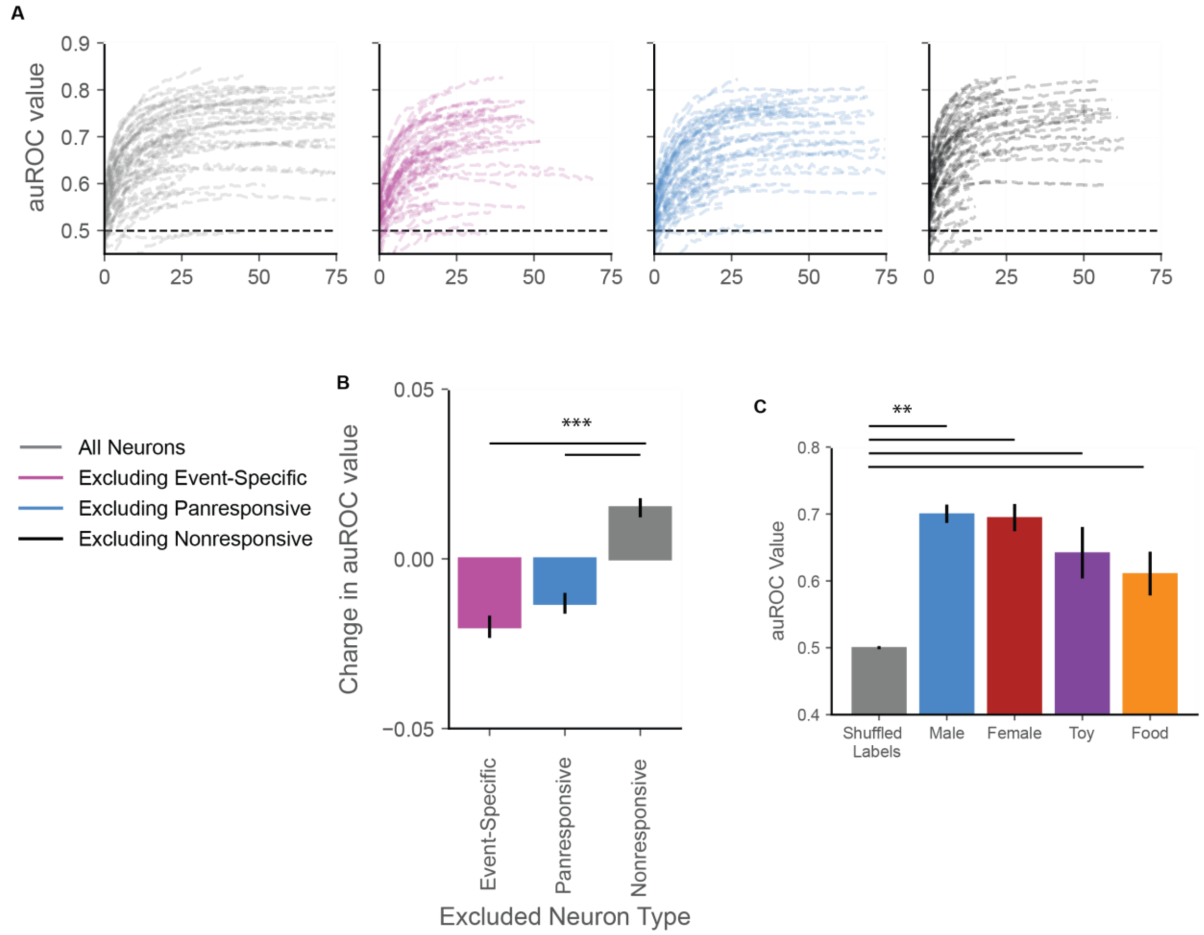

Fig. S9. Decoding interaction with stimuli from neural activity. Related to Figure3.
A) Accurate decoding of behavioral interaction with stimuli is highly dependent on the number of neurons used (each line represents a single event). Removing specific neuron types (i.e. event-specific, panresponsive or nonresponsive) mildly impacts the performance of the decoder. This is quantified in (B, **p< 0.01, event-specific: -0.02 ± 0.003, panresponsive: -0.01 ± 0.003, nonresponsive: 0.02 ± 0.003; mean ± SEM; N = 60 events, repeated-measures 1-way ANOVA).
C) Behavioral interaction can be accurately decoded from event types with any of the 4 stimuli (**p<0.01, shuffled(60): 0.50 ± 0.003, male(27): 0.70 ± 0.01, female(17): 0.69 ± 0.02, toy(8): 0.64 ± 0.04, food(8): 0.61 ± 0.03; event-type(N): mean ± SEM; Kruskal-Wallis test)

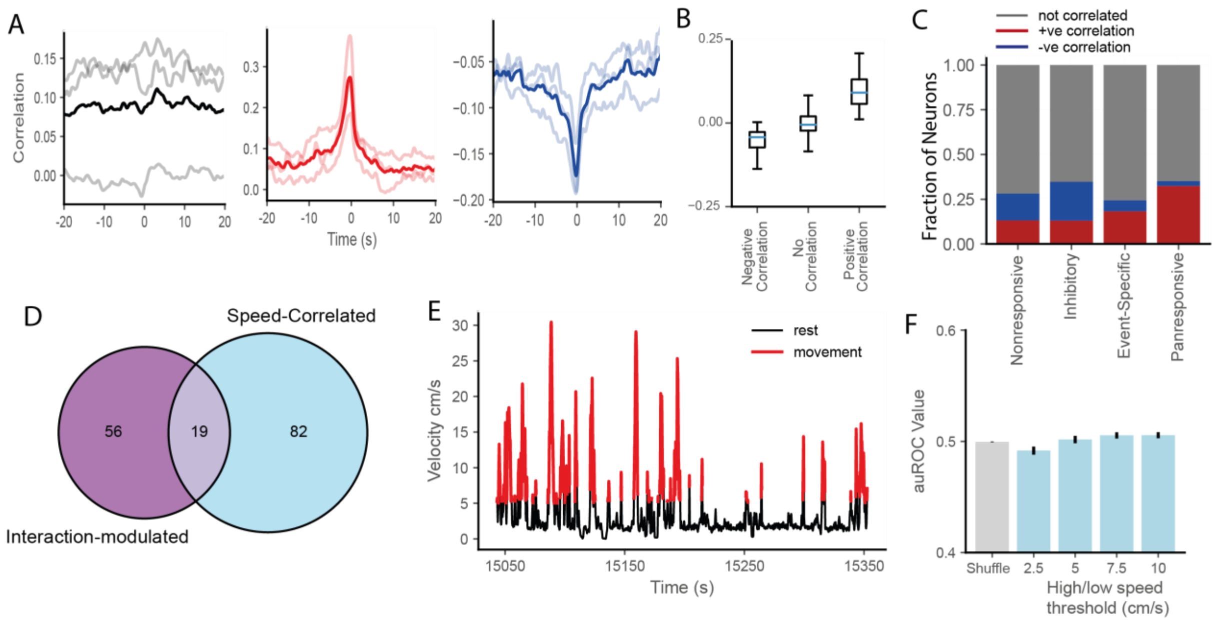

Fig. S10 BLA neurons show some correlation with speed but cannot decode movement. Related to Figure3.

A) Representative examples of BLA neurons either not correlated with speed (left), positively correlated (middle) or negatively correlated (right). The crosscorrelation of each unit with animal speed was calculated for baseline, event and post-event periods (lighter traces) and that average (darker trace) was used to determine the speed correlation. B) As a population, BLA units are only modestly correlated or anti-correlated with speed (negative: -0.04, none: -0.01, positive: +0.09, median correlation). C) Speed-correlated units are not over-represented by any one category of BLA neuron, however panresponsive units are more likely to be positively correlated with speed. D) Overall, there is little overlap between interaction-modulated units (see Figure3) and speed -correlated units. E) To test whether BLA firing can decode movement, behavioral data was divided into periods of rest or movement and tested using LDA decoding. F) Regardless of the exact threshold for rest versus movement, BLA firing activity was unable to decode movement. (p=0.09-0.92, high/low speed threshold categories compared to shuffle, N=60 events; repeated measures 1-way ANOVA)

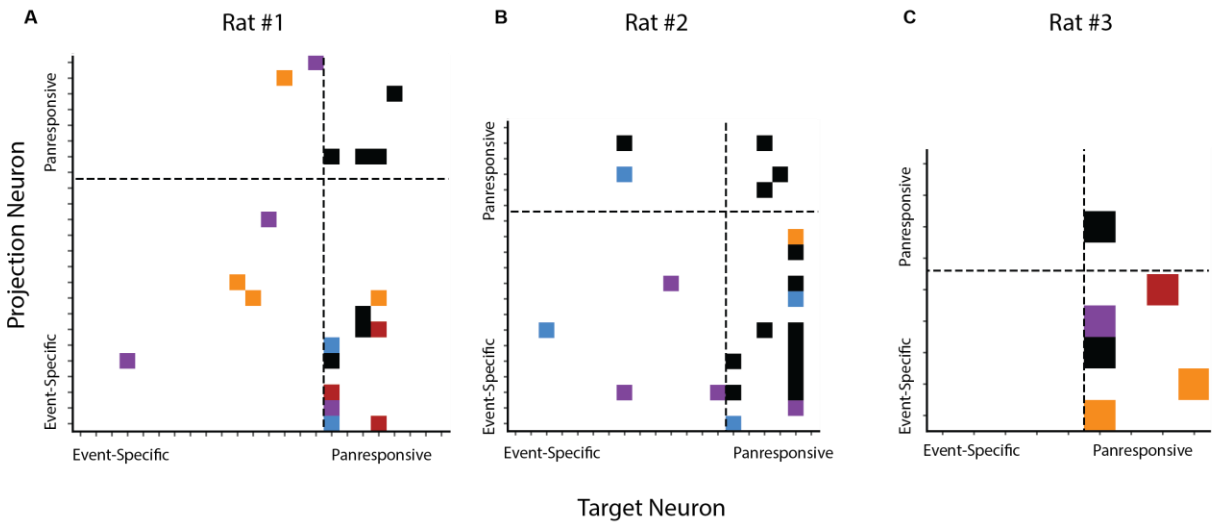

Fig. S11. Connectivity between event-specific and panresponsive neurons within individual rats. Related to Figure4.

A-C) Cross-correlated pairs of neurons within 3 separate rats during the first presentation of each stimulus. Colors represent whether the neuronal pair fired exclusively during a single presentation (red, blue, purple, orange represents female, male, toy, food respectively) or if the neuronal pair was active across multiple stimulus presentations (black, 2+ stimulus presentations). During the stimulus presentation the flow of neuronal information typically goes from event-specific to panresponsive neurons.
